# Biocatalyst–artificial metalloenzyme cascade based on alcohol dehydrogenase†
†Electronic supplementary information (ESI) available. See DOI: 10.1039/c8sc02371a


**DOI:** 10.1039/c8sc02371a

**Published:** 2018-08-14

**Authors:** Simone Morra, Anca Pordea

**Affiliations:** a Faculty of Engineering , University of Nottingham , University Park, NG7 2RD , Nottingham , UK . Email: anca.pordea@nottingham.ac.uk

## Abstract

Chemo-enzymatic cascades of enzymes with transition metal catalysts can offer efficient synthetic strategies, but their development is challenging due to the incompatibility between proteins and transition metal complexes. Rhodium catalysts can be combined with alcohol dehydrogenases to regenerate nicotinamide cofactors using formate as the hydride donor. However, their use is limited, due to binding of the metals to residues on the enzyme surface, leading to mutual enzyme and catalyst inactivation. In this work, we replaced the zinc from *Thermoanaerobacter brockii* alcohol dehydrogenase (TbADH) with Rh(iii) catalysts possessing nitrogen donor ligands, by covalent conjugation to the active site cysteine, to create artificial metalloenzymes for NADP^+^ reduction. TbADH was used as protein scaffold for both alcohol synthesis and the recycling of the cofactor, by combination of the chemically modified species with the non-modified recombinant enzyme. Stability studies revealed that the incorporation of the catalysts into the TbADH pocket provided a shielding environment for the metal catalyst, resulting in increased stability of both the recycling catalyst and the ADH. The reduction of a representative ketone using this novel alcohol dehydrogenase–artificial formate dehydrogenase cascade yielded better conversions than in the presence of free metal catalyst.

## Abstract 2

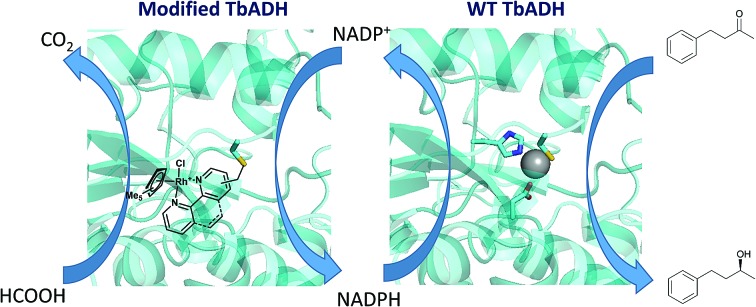
Alcohol dehydrogenase with a dual role: host for metal catalysts and enzyme for ketone reduction.

## 1 Introduction

Alcohol dehydrogenases (ADHs) are valuable biocatalytic tools for the conversion of ketones into chiral alcohols, providing a sustainable and efficient alternative to chiral catalysts based on transition metals.^1^ The main limitation to their practical application is the requirement of nicotinamide adenine dinucleotide cofactors (NADH or NADPH) as electron donors. The complexity of these structures, associated with high cost and reduced stability, precludes their use as stoichiometric reducing agents. Therefore, the continuous *in situ* regeneration of the cofactor is necessary for economically acceptable transformations with isolated enzymes. Current state of the art for NAD(P)H recycling is the use of a second catalyst to regenerate the cofactor at the expense of another reducing agent.^2^ Enzymatic recycling systems have been developed, inspired by the mechanisms of cofactor balance in living cells, but they are not universally applicable and may suffer from incompatibility between the two enzyme systems and complexity of product purification.^3^

Both chemical and electrochemical delivery of reducing potential have been investigated, as simpler and more robust alternatives to enzymatic methods.^2^–^6^ A very promising option are chemical catalysts based on Rh(iii), Ir(iii) and to a lesser extent Ru(ii), which use formate as a reducing agent.^7^–^12^ Although less active than formate dehydrogenase (turnover frequency ∼250 times lower for NADH formation for the state-of-the-art Rh complex),^3^,^13^ they are compatible with high temperatures and solvents, and avoid the complexity associated with a second enzyme. Additionally, organometallic complexes have been shown to recycle the simpler and less expensive synthetic NAD(P)H mimics.^14^,^15^ Sadler and co-workers also reported the formate-driven reduction of NAD^+^ to NADH catalysed by Ru(ii) arene complexes in living cells, albeit with reduced activity compared to the complexes in buffer.^16^

The main limitation of these synthetic regeneration catalysts is the incompatibility of the metal complexes with coordinating functionalities present in the mixture, such as accessible amine or thiol groups present in enzymes.^17^–^19^ Restricting the access of the metal species to the nucleophilic residues on the enzyme surface is required to solve this problem. Attempts to protect the catalysts by separately immobilising the enzyme and the metal complex have been shown to increase process stability, and offered opportunities for catalyst recycling.^4^,^6^ On the other hand, immobilisation of the catalysts resulted in decreased catalytic activity due to diffusion limitations.^20^

As an alternative to the immobilisation on solid support, artificial metalloenzymes are constructed by encapsulation of metal-based catalysts inside protein scaffolds, thus overcoming mass transfer limitations by keeping the catalytic moiety and the reagents in homogeneous phase. They have been explored as a synergistic method in catalyst design, by coupling the selectivity of enzymes with versatile, human-devised, catalytic functionality, to afford synthetic capabilities greater than the sum of the parts.^21^ Artificial metalloenzymes have been shown to facilitate a range of synthetic reaction chemistries, from reductions of carbon–carbon and carbon–heteroatom bonds, to oxygen insertion and carbon–carbon bond formation.^21^–^27^

The reduction of NAD(P)^+^ has also been achieved with metal catalysts incorporated into proteins, either by covalent linking to papain,^28^ or by supramolecular insertion using the well-established biotin–streptavidin system.^29^ In this context, Ward and co-workers reported that the protein host also provided a shielding environment for the metal catalyst against inhibitory species present in solution. This concept of “molecular compartmentalisation” has been demonstrated by an increased activity of the protein-bound compared to the free metal complex, when used *in situ* with various enzymes dependent on NAD(P)H or on NAD(P)H mimics.^30^–^32^ Whilst a range of cofactor-dependent biocatalysts have been employed in conjunction with artificial metalloenzymes, such as ene reductases, glucose dehydrogenase and 2-hydroxybiphenyl monooxygenase, a cascade reaction with alcohol dehydrogenases remains to be developed. This is most likely due to the ketone reduction activity of the most successful biotinylated Cp*Ir aminosulfonamide complexes reported so far, which would interfere with the ADH activity.^33^

**Fig. 1:**
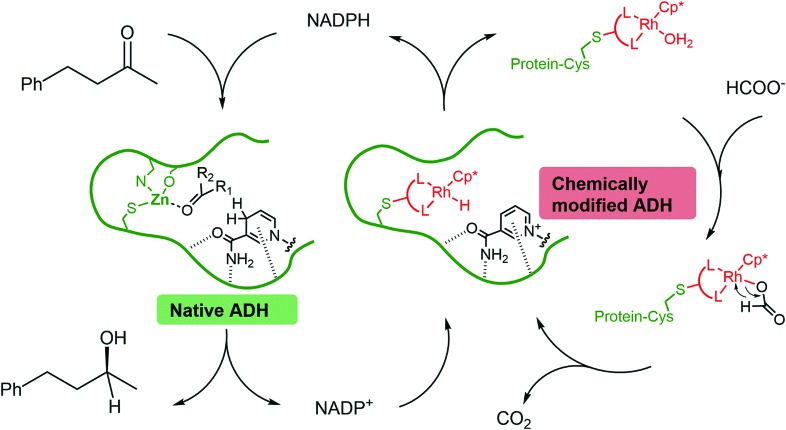
Reduction of a model ketone using a native–artificial enzyme cascade with the same alcohol dehydrogenase scaffold. L–L represents a bidentate ligand. The protein is represented in green, and the rhodium complex is represented in red.

In most cases, the design of artificial metalloenzymes relies on the availability of a bioconjugation site within the protein scaffold, whilst substrate-binding features are less important in the design. Yet, substrate binding in an enzyme active site can lead to exquisite control over the activation pathway, and thus to promiscuous reactivities, as demonstrated by Hyster and co-workers who used an NADPH-dependent reductase to catalyse radical formation and hydrogen atom transfer.^34^ Zinc-dependent alcohol dehydrogenases are well-suited to build artificial enzymes for NAD(P)^+^ reduction: they contain a metal-binding site, a hydrophobic substrate-binding pocket able to accommodate organic ligands, as well as the NAD(P)^+^ cofactor-binding pocket.^35^ In this study, we will present the development of hybrid catalysts for NADPH regeneration, by using a zinc-dependent ADH as host for metal complexes with demonstrated ability to reduce NAD(P)^+^. In the native ADH reaction, the reduction of NAD(P)^+^ takes place by hydride transfer from an alcohol hydride donor. We reasoned that the replacement of the alcohol by a metal hydride donor will provide the advantage of irreversible hydride transfer from formate to the cofactor. We hypothesise that the use of ADH as the protein scaffold for both alcohol synthesis (natural ADH) and the recycling of the cofactor (chemically modified ADH) will result in an increased robustness of a system where both reactions take place in the same mixture (Fig. 1). To test this hypothesis, the potential of the protein to prevent metal inactivation will be studied and the new artificial metalloenzymes will be compared with non-enzymatic cofactor regeneration catalysts in a synthetic cascade.

## 2 Results and discussion

### 2.1 Design and preparation of alcohol dehydrogenase mutants

**Fig. 2:**
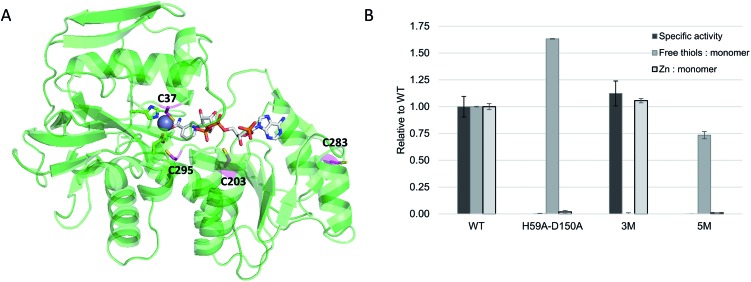
(A) Crystal structure of TbADH complexed to NADP^+^ (pdb ; 1YKF). Cysteine residues are highlighted in magenta, active site residues H59 and D150 are highlighted in green and the catalytic zinc ion is shown as a grey sphere; (B) engineering of TbADH for the covalent binding of small molecules into the active site. Absolute values for WT TbADH were as follows: specific activity (2-butanol oxidation coupled to NADP^+^ reduction, U = μmol min^–1^): 74 ± 7 U mg^–1^; free thiols: 0.98 ± 0.003 per monomer; Zn content: 0.64 ± 0.02 atoms per monomer.

ADH from the thermophilic species *Thermoanaerobacter brockii* (TbADH) was selected for bioconjugation to a metal complex catalyst. TbADH is a Zn-dependent bacterial ADH with a homotetrameric structure, in which the catalytic Zn is bound by residues C37, H59 and D150.^36^ Its distinguishing feature compared to other medium-chain Zn-dependent ADHs is the absence of a second, structural Zn-binding site. Wild-type TbADH reduces small aliphatic ketones with high enantioselectivity.^37^,^38^ Of particular interest is its NADP^+^ specificity, as well as its stability at relatively high temperatures (up to 65–86 °C)^39^ and in the presence of organic solvents.^37^,^40^ It was reasoned that its robustness would facilitate the introduction of organic ligands within the structure. Covalent anchoring to cysteine was selected as the method for the bioconjugation of metal catalysts. TbADH contains four cysteine residues per monomer (Fig. 2A). Two of these have already been covalently labelled with iodoacetic acid: C203 in the NADP^+^ binding pocket and C37 in the Zn-binding site, which could be readily modified in the absence of catalytic Zn. Previous work also showed that C203 could be mutated to serine without loss in enzyme activity.^41^

WT TbADH was overexpressed in *E. coli* and purified by affinity chromatography. The specific activity of this recombinant enzyme was comparable to the native enzyme.^39^ The accessibility of the cysteine residues and their reactivity towards 5,5′-dithio-bis(2-nitrobenzoic acid) (DTNB) was assessed by Ellman's assay, which indicated the availability of one thiol per enzyme monomer in WT TbADH, presumably C203 according to the structure and previous evidence.^41^ The triple mutant C203S-C283A-C295A (hereafter TbADH 3M), obtained by site-directed mutagenesis, was expressed and purified, showing similar specific activities compared to WT TbADH (Fig. 2B), thus suggesting that removal of these thiol groups does not significantly alter the native protein structure. This mutant showed no free thiol content, indicating that C37 was not available for modification in the presence of zinc. Zinc removal was achieved by further mutation of the Zn-coordinating residues to smaller alanine side chains. As expected and confirmed by ICP-MS analysis, double mutant H59A-D150A contained no zinc and its reaction with the Ellman's reagent showed the availability of 1.6 free thiols per monomer for covalent modification. Thus, a working TbADH scaffold containing the five mutations H59A-D150A-C203S-C283A-C295A (hereafter TbADH 5M) was prepared, devoid of zinc and containing C37 as the only reactive free thiol (Fig. 2B). It was hypothesized that a metal complex attached to C37 would fit within the Zn cavity, and in proximity of the nicotinamide C4 redox site.

### 2.2 Bioconjugation of metal complexes to TbADH

**Scheme 1:**
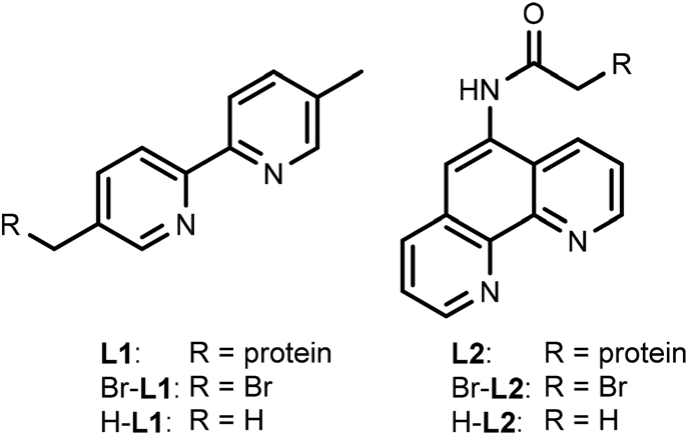
Chelate nitrogen donor ligands used in this study.

For this study, rhodium catalysts of the type [Cp*Rh^III^(N^N)(H_2_O)]^2+^ were selected, which were previously shown to recycle NAD(P)H with formate as the hydride donor (N^N = chelate nitrogen donor ligands).^7^,^9^ Bipyridine and phenanthroline ligands containing a bromide functionality (Scheme 1), and their corresponding metal complexes [Cp*Rh(Br-**L1**)Cl]Cl and [Cp*Rh(Br-**L2**)Cl]Cl, were prepared according to published procedures (see ESI†). Bromide was chosen as the alkylating functionality, because this allowed the design of the shortest possible anchor between the protein and the metal complex.

The bioconjugation of brominated ligands to TbADH was assessed by ESI-MS. Labelling of TbADH 5M with Br-**L2**, containing the bromo acetamide anchor, occurred readily at pH 7, yielding exclusively the mono-labelled protein. The modification with Br-**L1**, bearing an alkyl bromide functionality, required an increased pH of 8 to achieve more than 50% yield, and also resulted in unselective modification at a second site (see ESI†). Free thiols depletion after conjugation was assayed by the Ellman's assay and was in good agreement with the residual amount of free 5M observed by ESI-MS.

**Fig. 3:**
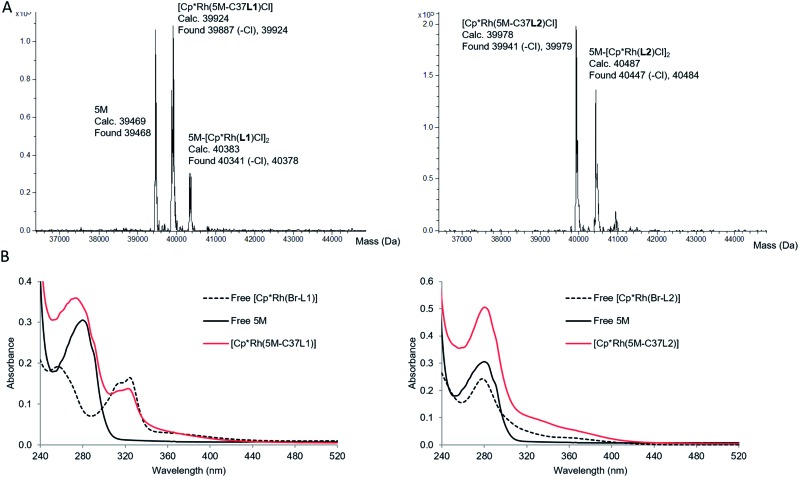
Characterisation of covalently labelled TbADH variants, [Cp*Rh(5M-C37**L1**)]^2+^ and [Cp*Rh(5M-C37**L2**)]^2+^ by (A) ESI-TOF mass spectrometry and (B) UV-Vis spectroscopy. UV-Vis analysis was performed in sodium phosphate buffer (100 mM, pH 7), at 10 μM concentration (determined by Bradford assay for the proteins, and by weight for the free rhodium complexes).

When the corresponding rhodium complexes were used, the alkylation at pH 7 was more specific than at pH 8 or 9, presumably because of unspecific rhodium binding to deprotonated surface residues at the higher pH. The optimum conditions for the formation of the mono-labelled proteins as the major compounds were obtained by incubating TbADH 5M with 4 equivalents of the metal complex in 100 mM Tris–HCl buffer at pH 7, for 1 h at room temperature. The proteins were subsequently purified by removing the excess labelling agent on PD-10 desalting columns. Using this procedure, [Cp*Rh(5M-C37**L1**)]^2+^ was obtained as a mixture with non-labelled 5M, while [Cp*Rh(5M-C37**L2**)]^2+^ was obtained as a mixture with the di-labelled protein (Fig. 3A). Additionally, MALDI-TOF analysis of the peptides obtained after protein digestion with chymotrypsin confirmed the covalent labelling at C37 (see ESI†). Protein concentration was determined by Bradford assay. Labelled and unlabelled protein samples of the same concentrations also showed similar SDS-PAGE band intensities, thus excluding interference of the metal complex with the assay. Inductively coupled plasma mass spectrometry (ICP-MS) analysis showed an average Rh : protein molar ratio of 0.5 : 1 in both cases. In subsequent experiments, unlabelled protein concentration was determined by Bradford, whilst labelled protein concentration was determined from the Rh ICP-MS analysis.

Characterisation of the labelled proteins by UV-Vis spectroscopy indicated the presence of the Rh complex in both protein samples. The UV-Vis spectrum of [Cp*Rh(5M-C37**L1**)]^2+^ showed an absorption band at 323 nm, which could not be observed in the non-labelled 5M mutant and was very similar to the absorption of free [Cp*Rh(Br-**L1**)]^2+^. Similarly, an increase in absorbance compared to 5M was observed for the bioconjugated [Cp*Rh(5M-C37**L2**)]^2+^ above 300 nm, indicating the presence of the rhodium–phenanthroline complex (Fig. 3B). The characteristic absorbance was very similar for the free and the bioconjugated metal complexes, indicating a similar primary coordination sphere around the rhodium centre.

To assess non-covalent binding of the catalysts outside the active site, the zinc-containing TbADH 3M mutant was treated with the non-brominated Rh catalysts [Cp*Rh(H-**L1**)Cl]Cl and [Cp*Rh(H-**L2**)Cl]Cl. Binding of 0.5 equiv. of rhodium to the protein was still observed by ICP-MS, although at a different site from C37, as indicated by the almost completely retained ketone reduction activity of the 3M mutant (data not shown). This suggested that di-labelling of the protein was due to rhodium coordination, rather than covalent modification of residues other than cysteines. Purification by ultrafiltration, extensive dialysis or by passing through desalting columns could not remove the Rh complex, suggesting its strong binding to the protein. Literature has already reported the binding of up to 4 equivalents of [Cp*Rh(bipy)(H_2_O)]^2+^ to the thermostable TADH, potentially to surface histidine and cysteine residues, without influencing the catalytic activity of the enzyme.^17^ Here, we observed a similar propensity of TbADH to unspecifically bind rhodium complexes. Given that formation of a single TbADH species covalently bound to one rhodium complex could not be achieved, we proceeded with the evaluation of the mixture characterised above.

### 2.3 Formate dehydrogenase activity of artificial metalloenzymes

The ability of the new artificial metalloenzymes to reduce NADP^+^ using formate as the hydride source was evaluated next, by following the increase of absorbance at 340 nm, due to the formation of NADPH (Table 1). Interestingly, a lag phase could be observed for about 20–30 seconds at the beginning of the reactions, during which the enzyme activity was negligible, after which time the reaction started (see ESI†). This might be due to the shielding effect of the protein matrix, slowing down the initial attachment of either formate or NADP^+^ to the catalyst. The turnover frequency (TOF) at 50 °C was calculated from the reaction rate measured during linear absorbance increase. TOF was defined as the quantity of NADPH formed per unit of time (calculated using *ε*_340_ = 6220 M^–1^ cm^–1^), and divided by the quantity of the rhodium catalyst (determined by ICP-MS).

**Table 1:** Reduction of NADP^+^ by Cp*Rh complexes in the presence of formate^*a*^

Entry	Catalyst	Substrate	TOF_Rh_ (h^–1^)
1	[Cp*Rh(Br-**L1**)Cl]Cl	NADP^+^	248
2	[Cp*Rh(Br-**L2**)Cl]Cl	NADP^+^	207
3	[Cp*Rh(5M-C37**L1**)]^2+^	NADP^+^	59
4	[Cp*Rh(5M-C37**L2**)]^2+^	NADP^+^	74
5	[Cp*Rh(5M-C37**L1**)]^2+^	NAD^+^	36

^*a*^Catalysis conditions: NAD(P)^+^ (0.15 mM), sodium formate (500 mM), Rh catalyst (12.5 μM with artificial metalloenzymes; 25 μM with free catalyst) in buffer (sodium phosphate, 100 mM, pH 7) at 50 °C. The reduction rate was measured by monitoring the absorbance at 340 nm.

The activity of the brominated free catalysts [Cp*Rh(Br-**L1**)Cl]Cl and [Cp*Rh(Br-**L2**)Cl]Cl was similar to previous literature results (entries 1–2).^7^,^13^ When covalently anchored to TbADH, the rhodium catalysts were less active than in their free form (entries 3–4), however their activities were comparable with previously published artificial metalloenzymes for NAD^+^ reduction.^28^,^30^ Such a decrease in activity between free and protein-embedded catalysts has previously been observed for the NADH regeneration with biotinylated Cp*Ir complexes incorporated into streptavidin.^30^ Some specificity for NADP^+^*vs.* NAD^+^ was observed with the bioconjugated Rh–bipyridine complex (Table 1, entry 5), but not when the phenanthroline ligand was used. This result does not reflect the NADP^+^-specificity of WT TbADH (specific activity is approx. 120 times higher with NADP^+^ than with NAD^+^, see ESI†). This is likely due to the rhodium hydride in the artificial metalloenzyme being more exposed than the zinc-bound alcoholate catalytic species in the wild-type enzyme, and possibly rearranging the cofactor binding.

### 2.4 Compatibility between bioconjugated Rh catalysts and TbADH

**Fig. 4:**
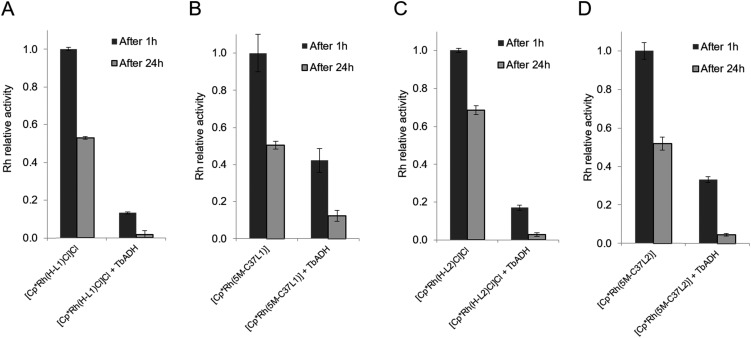
Inactivation of rhodium catalysts by TbADH. (A) Non-conjugated [Cp*Rh(H-**L1**)Cl]Cl; (B) bioconjugated [Cp*Rh(5M-C37**L1**)]^2+^; (C) non-conjugated [Cp*Rh(H-**L2**)Cl]Cl; (D) bioconjugated [Cp*Rh(5M-C37**L2**)]^2+^. Rhodium catalysts (10 μM) were incubated with 1 equiv. WT TbADH (10 μM) in sodium phosphate buffer (100 mM, pH 7) at 50 °C. Catalyst activity was determined by monitoring NADPH formation at 340 nm in the presence of formate (100 mM) at 50 °C. In the absence of TbADH after 1 h heating, TOF_Rh_ were as follows: 147 ± 1 h^–1^ for [Cp*Rh(H-**L1**)Cl]Cl; 28 ± 1 h^–1^ for [Cp*Rh(5M-C37**L1**)]^2+^; 112 ± 2 h^–1^ for [Cp*Rh(H-**L2**)Cl]Cl; 38 ± 1 h^–1^ for [Cp*Rh(5M-C37**L2**)]^2+^.

The mutual inactivation between the Rh-based catalysts and wild-type TbADH was assessed after incubation at 50 °C for 1 h and for 24 h (Fig. 4). In the presence of 1 equiv. TbADH (Rh : TbADH molar ratio = 1 : 1), the free catalysts lost most of their activity after incubation at 50 °C. This is in line with previously published data by Hollmann and co-workers, who demonstrated complete inactivation of up to 4 equivalents of [Cp*Rh(Bipy)(H_2_O)]^2+^ after incubation for 1 h with TADH from *Thermus* ATN1 species, suggesting the presence of four Rh binding sites within this enzyme.^17^ In our work, some residual Rh activity was still observed after 1 h incubation, suggesting that the inactivating effect on the rhodium complex was less significant with TbADH than with TADH. However, the activity of the complexes after incubation with TbADH was far lower than in the absence of the enzyme.

When the bioconjugated catalysts were incubated with 1 equiv. TbADH, an increased stability was observed compared to the free complexes, with 42% and 33% residual activity observed after 1 h incubation for [Cp*Rh(5M-C37**L1**)]^2+^ and [Cp*Rh(5M-C37**L2**)]^2+^, respectively. This observation confirms that the protein scaffold acts as a shielding environment around the Rh catalysts, protecting these from deactivation by unspecific interactions with residues on the surface of the enzyme. Higher Rh/TbADH ratios resulted in increased relative activity of the complexes (see ESI†).

**Fig. 5:**
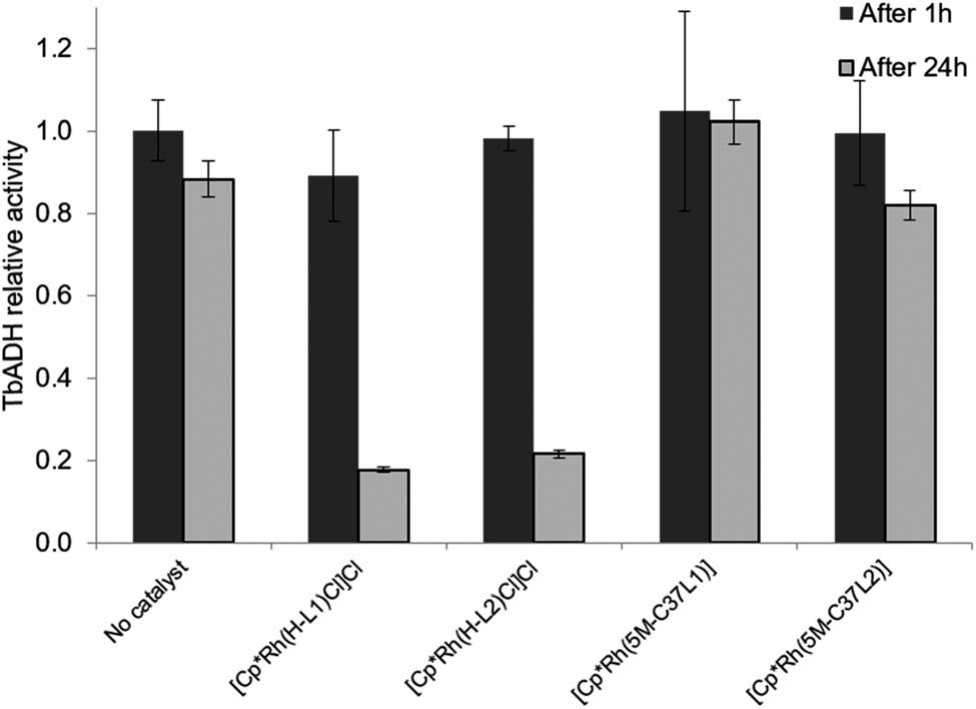
Effect of the free and bioconjugated rhodium catalysts on 2-butanone reduction by WT TbADH. WT TbADH (1 μM) was incubated with the corresponding Rh catalyst (5 μM) in sodium phosphate buffer (100 mM, pH 7). In the absence of the Rh catalyst, the specific activity of WT TbADH (2-butanone reduction coupled to NADPH oxidation) after 1 h heating was 4.95 ± 0.37 U mg^–1^.

The beneficial effect of bioconjugation was even more prominent in the case of TbADH deactivation by the Rh catalysts. When exposed to 5 equivalents of Rh catalyst for 1 h, TbADH activity in the reduction direction remained relatively constant, irrespective of the type of catalyst used. However, when the incubation time was 24 h, the activity of TbADH decreased drastically when free Rh catalyst was present, but remained >80% in the presence of the artificial metalloenzymes (Fig. 5). Previous work by Steckhan and co-workers suggested no inhibition of TbADH after incubation with 0.5 mM [Cp*Rh(bipy)(H_2_O)]Cl_2_, however their experiments were performed at 30 °C and did not state the enzyme concentration.^8^ On the other hand, Hollmann and co-workers have previously shown that inactivation of TADH by the same catalyst occurred upon incubation with more than 4 equiv. of Rh. Moreover, inactivation was pH-dependent, with 80% activity being retained after 1 h incubation at pH 8 and room temperature, and only 25% of the TADH activity remaining after incubation at pH 6 with 100-fold Rh molar excess.^17^ In our hands, increased inactivation of TbADH by the Rh(iii) complex can be observed at 50 °C, but can be avoided by physically separating the complex from the enzyme surface, by encapsulation into the protein.

### 2.5 Cofactor recycling cascades

Encouraged by the beneficial effect of bioconjugation on preventing mutual inactivation between TbADH and the Rh regeneration catalysts, we developed an ADH–hybrid catalyst cascade for the reduction of 4-phenyl-2-butanone. In this system, TbADH was used as the same protein scaffold for both ketone reduction and NADPH regeneration driven by formate (see Fig. 1).

The reduction of 4-phenyl-2-butanone by TbADH was tested at 50 °C for 24 h, using 0.5% (mol) of either free or bioconjugated NADPH regeneration catalysts (see Fig. 1). Given the much lower turnover frequencies of Rh catalysts (∼0.02 s^–1^) compared to TbADH (∼2 s^–1^ for 2-butanone, data not shown), the amount of TbADH necessary to perform the transformation should be orders of magnitude lower than the amount of Rh. However, in line with stability studies, TbADH inactivation occurred at concentrations <1 μM, which resulted in the use of an optimised ratio of 25 μM Rh and 1.5 μM WT TbADH. The extent of inactivation was lower when bioconjugated catalysts were used (see ESI†). Although the initial activity of the free Rh catalysts for NADP^+^ reduction was higher (see Table 1), the bioconjugated catalysts had better overall performance for the reduction of the ketone substrate, with 18–20% increase in conversion being observed (Table 2). This is likely to be due to the increased stability of both the ketone reduction, and the NADPH regeneration catalysts present in the same reaction vessel over 24 h. The enantioselectivity of the reaction was not affected by the presence of the catalyst, in either free or bioconjugated form (>99% ee (*S*) was obtained in all cases). Ketone reduction by the artificial metalloenzymes was very slow, and could only be detected at rhodium concentrations much higher than those used in the regeneration experiments (TOF 0.1–0.2 h^–1^). This is in line with previous reports, where a very low turnover was observed with the [Cp*Rh(bipy)(H_2_O)]^2+^ catalyst, compared to enzymatic ketone reduction, under aqueous conditions at neutral pH.^8^,^42^

**Table 2:** Reduction of 4-phenyl-2-butanone using TbADH and Rh-based NADPH regeneration catalysts^*a*^

Entry	Regeneration catalyst	Conversion (%)
1	—	0
2	[Cp*Rh(5M-C37**L1**)]^2+^	67
3	[Cp*Rh(H-**L1**)Cl]Cl	57
4	[Cp*Rh(5M-C37**L2**)]^2+^	71
5	[Cp*Rh(H-**L2**)Cl]Cl	58

^*a*^Catalysis conditions: 4-phenyl-2-butanone (5 mM), NADP^+^ (0.5 mM), sodium formate (500 mM), Rh catalyst (25 μM), WT TbADH (1.5 μM) in buffer (sodium phosphate, 100 mM, pH 7) at 50 °C. Conversion and enantioselectivities were determined after 24 h by chiral HPLC, using a CHIRALCEL OD column. Conversion are presented as averages of triplicate runs, and standard deviations were <1%. Enantioselectivities were >99% ee in favour of the (*S*)-enantiomer, in all cases.

## 3 Experimental

### 3.1 Bioconjugation of metal complexes to TbADH

The gene encoding for wild-type TbADH bearing an N-terminal StrepTagII, cloned in the expression vector pET21a, was obtained from Biomatik (Ontario, Canada). Mutagenesis was performed using the QuikChange Lightning Site-Directed Mutagenesis Kit (Agilent Technologies), following manufacturer's instructions. Recombinant overexpression was carried out in *E. coli* BL21(DE3). Wild-type TbADH and mutants H59A-D150A-C203S-C283A-C295A (5M) and C203S-C283A-C295A (3M) were routinely characterized by SDS-PAGE, enzymatic activity, protein concentration, ICP-MS to determine metal content and Ellman assay to determine availability of thiol groups. The wild-type TbADH sequence, primers used for mutagenesis and details of protein expression, purification and characterisation can be found in the ESI.† Ligands Br-**L1** and Br-**L2**, and metal complexes [Cp*Rh(Br-**L1**)Cl]Cl and [Cp*Rh(Br-**L1**)Cl]Cl were synthesized according to the literature.

Covalent conjugation of complexes [Cp*Rh(Br-**L1**)Cl]Cl and [Cp*Rh(Br-**L1**)Cl]Cl to TbADH 5M was performed in 100 mM Tris–HCl pH 7, by incubating 4-fold molar excess of the brominated Rh catalysts with TbADH 5M, for 1 h at room temperature. Unbound catalyst was removed by desalting through a PD-10 column (GE Healthcare), followed by concentration by ultrafiltration with Vivaspin 6 (10 kDa MWCO, Sartorius). The flowthrough was routinely tested for the absence of catalytic activity and Rh content, thus supporting the absence of free catalyst in the artificial metalloenzyme preparations. UV-visible absorbance spectra were acquired in 100 mM sodium phosphate pH 7 with a Cary 8454 spectophotometer (Agilent Technologies). Rh content was determined by inductively coupled plasma mass spectrometry (ICP-MS) with a Thermo Fisher iCAP-Q instrument, after digestion of the protein in nitric acid (trace metal grade, Fisher Scientific) as previously described.^43^ Mass spectrometry analysis was performed by electrospray ionisation time-of-flight (ESI-TOF) in a Bruker Impact II spectrometer. Before analysis, the protein samples were desalted in pure water, concentrated up to ∼5 mg mL^–1^ and then mixed with 1 volume of 0.1% formic acid in acetonitrile.

### 3.2 Formate-driven NAD(P)^+^ reduction assays

The reduction rate was measured by monitoring the absorbance at 340 nm (*ε* = 6220 M^–1^ cm^–1^) with a Shimadzu UV-2600 spectrophotometer, equipped with CPS-100 temperature controller. Reactions were performed as previously described,^7^ in 100 mM sodium phosphate, 500 mM sodium formate pH 7 at 50 °C. NAD(P)^+^ concentration was 0.15 mM. Control experiments were carried out in the absence of any catalyst or in the absence of formate.

### 3.3 Stability assays

Metal concentration was determined by weight (free catalysts) or by ICP-MS (artificial metalloenzyme). Stability tests were adapted from previously reported mutual inactivation experiments with [Cp*Rh(Bipy)H_2_O]^2+^ and TADH.^17^ To test the inactivation of the Rh catalyst by TbADH, 10 μM Rh catalyst (non-brominated complex, or corresponding artificial metalloenzyme) was incubated with 10 μM TbADH in 100 mM sodium phosphate pH 7 at 50 °C. Samples were taken after 1 h or 24 h and the residual activity was assayed spectrophotometrically after the addition of sodium formate (100 mM final concentration) and NADP^+^ (0.15 mM final concentration).

To test the inactivation of TbADH by the Rh catalyst, 1 μM TbADH was incubated with 5 μM Rh catalyst (non-brominated catalyst, or corresponding artificial metalloenzyme) in 100 mM sodium phosphate pH 7 at 50 °C. Samples were taken after 1 h or 24 h and the residual activity was assayed spectrophotometrically after the addition of 2-butanone (50 mM final concentration) and NADPH (0.2 mM final concentration).

### 3.4 NADPH recycling assays

The assay was performed by adapting previous literature protocols.^8^ The reaction mixture (250 μL) contained 4-phenyl-2-butanone (5 mM), TbADH (1.5 μM), NADP^+^ (0.5 mM), Rh catalyst (non-brominated catalyst, or corresponding artificial metalloenzyme, 25 μM) and sodium formate (500 mM), in 100 mM sodium phosphate pH 7. The mixture was incubated for 24 h at 50 °C, then cooled to room temperature, extracted with 1 volume of hexane, dried over Na_2_SO_4_ and analysed by chiral HPLC. The analysis was performed with an Agilent 1220 Infinity equipped with a CHIRALCEL® OD column (4.6 mm × 250 mm; particle size 10 μm). The separation was isocratic at 1 mL min^–1^, using an isopropanol/hexane mixture 6/94 (v/v). The detection wavelength was 215 nm. Quantification was achieved by comparing peak area against standard solutions. Omitting any of the component from the reaction mixture resulted in no conversion.

## Conclusions

Alcohol dehydrogenase was for the first time used as a protein host for the design and creation of artificial metalloenzymes. Cp*Rh(iii) complexes of bipyridine and phenanthroline bearing an electrophilic moiety were covalently anchored to thermostable TbADH devoid of active site zinc and containing a single cysteine in the protein sequence. The resulting conjugated complexes were shown to catalyse the reduction of NADP^+^ at 50 °C. The mutual inactivation between wild-type TbADH and the ADH-based artificial metalloenzymes was evaluated and showed an increased stability of both the rhodium catalysts and the wild-type TbADH, when bioconjugated species were used instead of free complexes. The ADH-based artificial metalloenzymes were successfully combined in cofactor recycling cascades with wild-type TbADH, and afforded higher conversions compared to the free catalysts for the enantioselective reduction of 4-phenyl-2-butanone. The activity of these artificial metalloenzymes remains 2 orders of magnitude lower than the activity of recently discovered NADP^+^-dependent native formate dehydrogenase systems (0.02 s^–1^*vs.* up to 4.8 s^–1^).^44^ However, their compatibility with high temperatures and neutral pH makes them interesting candidates for further development of regeneration methods with bioconjugated transition metal complexes. Further research is in progress to optimise the conjugation site and to improve the binding specificity of rhodium complexes to TbADH. Additional metals such as Ru and Ir, as well as different ligand architectures will also be considered, given their reported ability to reduce nicotinamide cofactors.

## Conflicts of interest

There are no conflicts to declare.

## Supplementary Material

Supplementary information
