# Supplementary material for: Biocatalyst–artificial metalloenzyme cascade based on alcohol dehydrogenase
Source: Chem Sci. 2018 Aug 14;9(38):7447–54. doi: 10.1039/c8sc02371a (PMC6180310; doi:10.1039/c8sc02371a)
Supplement: Supplementary file 1 [file SC-009-C8SC02371A-s001.pdf]

Electronic Supplementary Material (ESI) for Chemical Science.
This journal is © The Royal Society of Chemistry 2018

Electronic Supplementary Information to the article

Biocatalyst – artificial metalloenzyme cascade based on alcohol dehydrogenase

Simone Morra[a,b] and Anca Pordea[a*]

[a] Faculty of Engineering, University of Nottingham, Nottingham, United Kingdom

[b] Present address: Inorganic Chemistry Laboratory, Department of Chemistry, University of Oxford, Oxford OX1 3QR, United Kingdom

Correspondence: Dr. Anca Pordea, Faculty of Engineering, University of Nottingham, University Park, NG7 2RD, Nottingham, United Kingdom.

E-mail: anca.pordea@nottingham.ac.uk

1. General considerations

All chemicals and reagents were purchased from Sigma Aldrich (unless otherwise stated) and were used without further purification. All solutions were prepared in ultrapure water (Milli-Q®, Millipore). All measurements were performed at least in triplicate.

2. Synthesis

NMR spectra were recorded at 298 K using Bruker instruments AV(III)400, AV400, DPX400 (400 MHz for ^{1}H, and 100 MHz for ^{13}C); or Bruker DPX300 (300 MHz for ^{1}H, and 75 MHz for ^{13}C). Chemical shifts (δ) are denoted in parts per million (ppm), referenced to the residual deuterated solvent quoted in text, as internal standard. Coupling constants J are denoted in Hz, with multiplicity of each signal designated with the following abbreviations: s, singlet; d, doublet; t, triplet; dd doublet of doublets; m, multiplet. Mass spectra were recorded on a Bruker MicroTOF 61 mass spectrometer using electrospray ionization (ESI^{+}, positive mode). Flash chromatography was carried out using Davisil silica 60 Å, with the eluent specified.
5-bromomethyl-5'-methyl-2,2'-bipyridine[1] and 2-bromo-N-(1,10-phenanthrolin-5-yl)acetamide hydrobromide[2] were synthesised following previously described protocols. 5,5'-dimethyl-2,2'-bipyridine, and all reagents and solvents were obtained from commercial sources in analytical purity.

Cp*Rh (5-bromomethyl-5'-methyl-2,2'-bipyridine) chloride, [Cp*Rh(Br-L1)Cl]Cl.

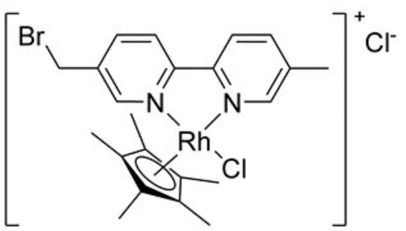

This compound was synthesized by adapting a previously described protocol.[3]
Working under nitrogen, pentamethylcyclopentadienyl rhodium(III) chloride dimer (100 mg, 0.162 mmol, 1 eq) was suspended in methanol (2 mL). To this suspension was added a solution of 5-bromomethyl-5'-methyl-2,2'-bipyridine (85 mg, 0.324 mmol, 2 eq) of in methanol (1 mL).

The mixture immediately turned into a yellow/orange clear solution, indicating the formation of the rhodium-bipyridine complex. The desired product was precipitated by addition of diethylether, collected by filtration and dried, to obtain a yellow powder (119 mg, 64%).

ESI^{+}-MS. calc. for $C_{22}H_{26}BrClN_2Rh$ $[M]^+$ 535.0017; found 535.0021.

CHN analysis. Product obtained as a mixture of Cl / Br salts 3 / 1 (mol). $C_{22}H_{26}Br_{1.25}Cl_{1.75}N_2Rh$ requires C, 45.30 %; H, 4.49 %; N, 4.80 %; found C, 45.37 %; H, 4.57 %; N, 4.69 %.

^{1}H NMR (300 MHz, $CDCl_3$). δ (ppm) 8.94 (1H, d, J = 5.84 Hz); 8.86 (1H, m); 8.82 (1H, m); 8.50 (1H, d, J =10.74 Hz); 8.27 (1H, d, J = 7.16 Hz); 8.02 (1H, d, J = 7.16 Hz); 4.90 (2H, s); 2.53 (3H, s); 1.73 (15H, d, J = 10.93 Hz).

^{13}C NMR (75 MHz, $CDCl_3$). δ (ppm) 154.10, 154.02, 151.81, 151.75, 151.36, 151.27, 150.89, 150.81, 141.48, 140.60, 139.32, 139.21, 138.11, 137.99, 125.25, 125.20, 124.86, 124.80, 97.85, 97.55, 97.37, 97.26, 42.37, 19.02, 9.72, 9.44.

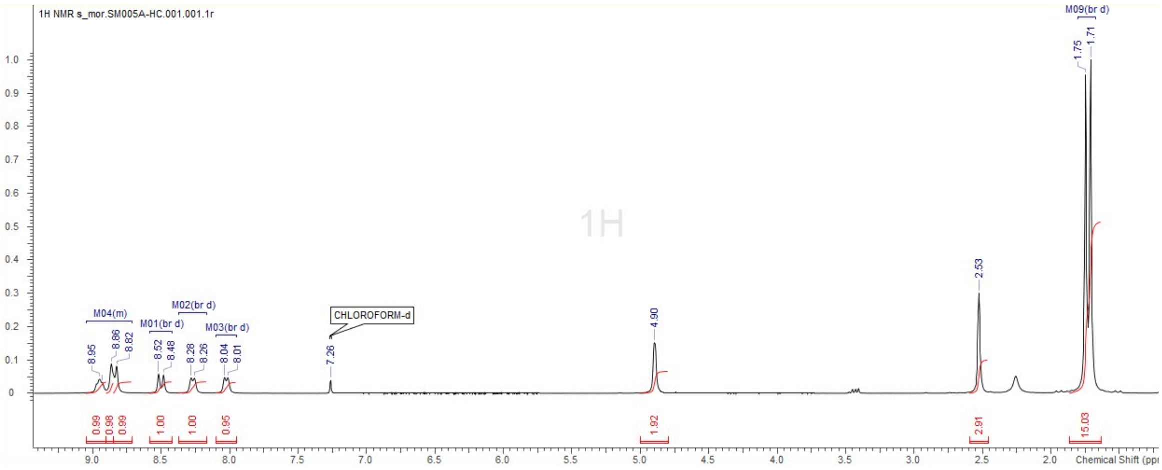

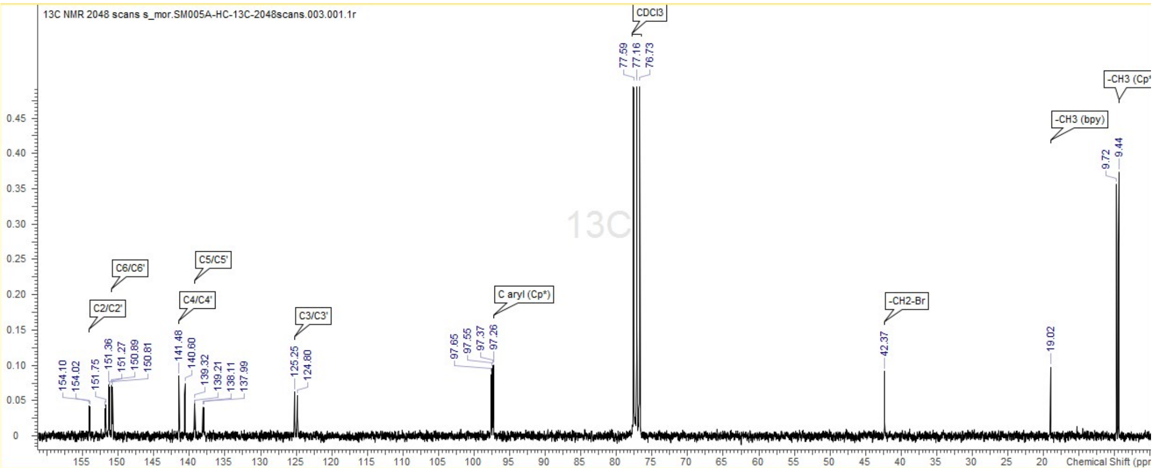

Cp*Rh (2-bromo-*N*-(1,10-phenanthrolin-5-yl)acetamide) chloride, [Cp*Rh(Br-L2)Cl]Cl.

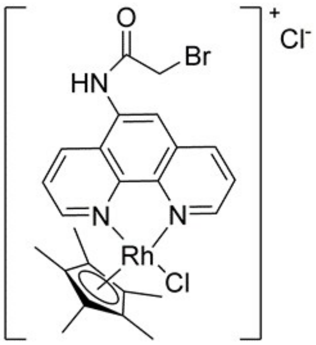

Working under nitrogen, pentamethylcyclopentadienyl rhodium(III) chloride dimer (100 mg, 0.162 mmol, 1 eq) was suspended in methanol (2 mL). To this suspension was added a solution of 2-bromo-N-(1,10-phenanthrolin-5-yl)acetamide hydrobromide (128 mg, 0.324 mmol, 2 eq) in methanol (1 mL). Triethylamine (69 µL, 49 mg, 0.485 mmol, 3 eq) was added and the mixture immediately turned into a yellow/orange clear solution, indicating the formation of the rhodium-phenanthroline complex. The product was precipitated by addition of diethylether; to remove residual traces of triethylamine, the solid was re-dissolved in 2 mL methanol and precipitated a second time with diethylether, to yield a yellow powder (66 mg, 33%). The compound was obtained as a Cl/Br mixture (1/3 ratio), as identified by ^{1}H NMR.

ESI^{+}-MS. calc. for $C_{24}H_{25}BrClN_{3}ORh$ $[M]^{+}$ 587.9919; found 587.9910.

CHN analysis. Product obtained as a mixture of Cl / Br salts 1 / 3 (mol). $C_{24}H_{25}Br_{1.75}Cl_{1.25}N_{3}ORh$ requires C, 43.77 %; H, 3.83 %; N, 6.38 %; found C, 43.24 %; H, 3.82 %; N, 6.04 %.

^{1}H NMR (400 MHz, $CDCl_3$). δ (ppm) 9.96 (1H, d, J = 8.33 Hz); 9.08 (1H, d, J = 4.97 Hz); 8.98 (1H, d, J = 5.12); 8.71 (1H, d, J = 4.02 Hz); 8.50 (1H, d, J = 8.08 Hz); 8.12 (1H, dd, J = 8.55, 5.19 Hz); 7.92 (1H, dd, J= 8.26, 5.04); 4.89 & 4.70 (2H, dd & s); 1.82 (15H, s).

^{13}C NMR (100 MHz, CD_3OD). δ (ppm) 169.13, 154.14, 153.45, 147.01, 145.05, 139.98, 136.11, 134.32, 131.56, 128.55, 128.22, 127.68, 122.89, 99.22, 99.14, 43.82, 9.30, 9.00.

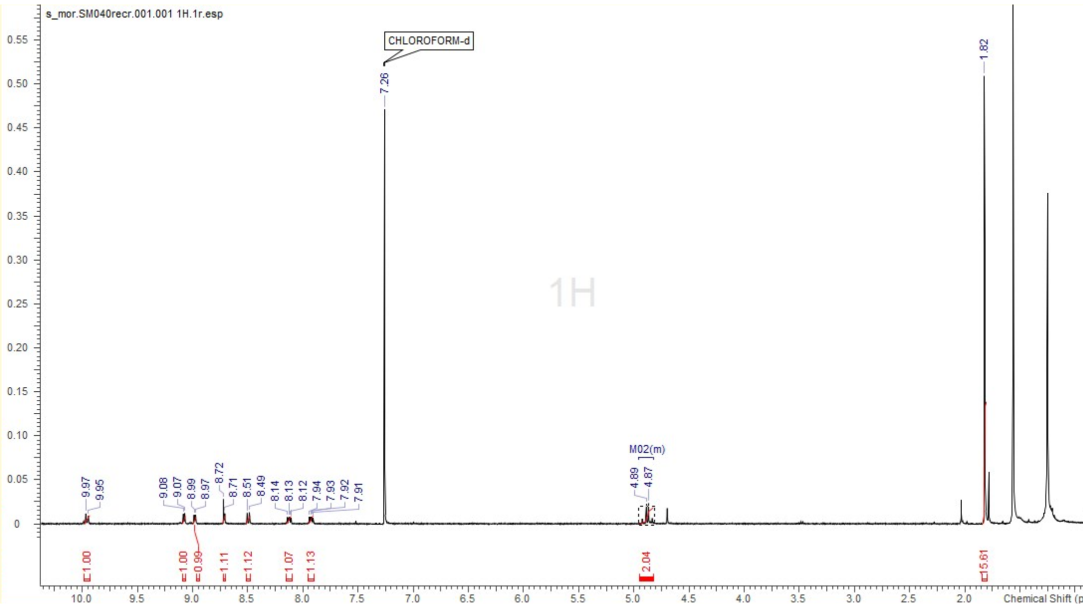

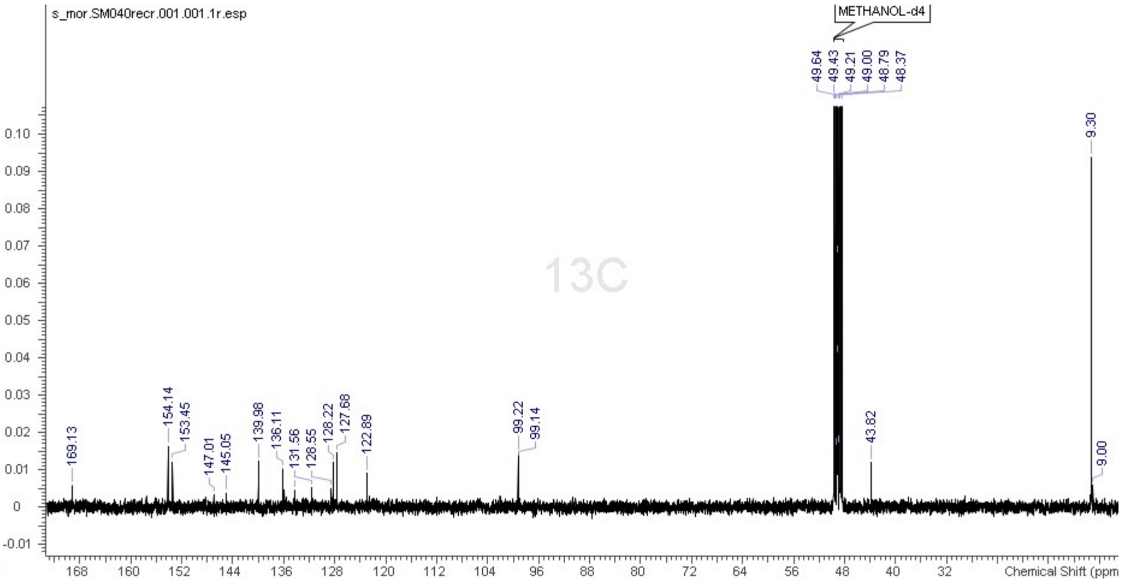

***N*-(1,10-phenanthrolin-5-yl)acetamide, H-L2.**

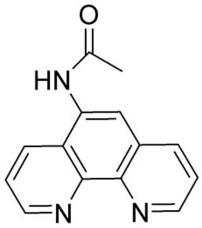

This compound was synthesized by adapting the protocol used for the brominated derivative. To 60 mL anhydrous $CHCl_3$ was added 390 mg (2 mmol, 1 eq) 1,10-phenantroline-5-amine, in a 100 mL two-necked flask, under nitrogen atmosphere. To this solution was slowly added acetyl

bromide (178 µL, 294 mg, 2.4 mmol, 1.2 eq). The reaction mixture was heated under reflux overnight and the resulting suspension was filtered. The solid was recrystallized from hot methanol, yielding the HBr salt of the desired compound as a yellow/orange solid (387 mg). The salt was dissolved in 10 mL of aqueous 1M NaOH and extracted 4 times with ethyl acetate. The organic phase was dried over Na_2SO_4 and the solvent evaporated, yielding the free base of the desired compound as a pale yellow powder (92 mg, 17%).

ESI$^+$-MS. calc. for $C_{14}H_{12}N_3O$ $[M + H]^+$ 238.0975; found 238.0980.

^{1}H NMR (400 MHz, DMSO-d$_6$). δ (ppm) 10.14 (1H, s, NH); 9.13 (1H, dd, J = 4.24, 1.61 Hz); 9.03 (1H, dd, J = 4.24, 1.75 Hz); 8.63 (1H, dd, J = 8.48, 1.46 Hz); 8.44 (1H, dd, J = 8.18, 1.68 Hz); 8.18 (1H, s); 7.82 (1H, dd, J = 8.33, 4.24); 7.73 (1H, dd, J = 8.04. 4.24); 2.25 (3H, s).

^{13}C NMR (100 MHz, DMSO-d$_6$). δ (ppm) 169.44, 149.82, 149.25, 145.84, 143.76, 135.76, 131.85, 131.64, 128.08, 124.56, 123.53, 122.79, 119.81, 23.59.

Cp* Rh (5,5'-dimethyl-2,2'-bipyridine) chloride, [Cp*Rh(H-L1)Cl]Cl.

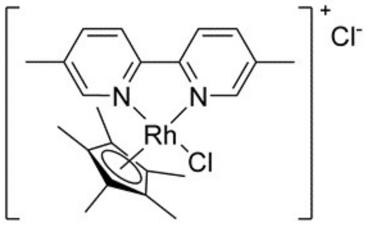

This compound was synthesised by adapting a previously described protocol.[3] Working under nitrogen, pentamethylcyclopentadienyl rhodium(III) chloride dimer (100 mg, 0.162 mmol, 1 eq) was suspended in methanol (2 mL). To this suspension was added a solution of 5,5'-dimethyl-2,2'-bipyridine (60 mg, 0.324 mmol, 2 eq) in methanol (1 mL). The mixture immediately turned into a yellow/orange clear solution, indicating the formation of the rhodium-bipyridine complex. The desired product was precipitated by addition of diethylether, collected by filtration and dried, to obtain a yellow powder (103 mg, 65%).

ESI$^+$-MS: calc. for $C_{22}H_{27}ClN_2Rh$ $[M]^+$ 457.0912; found 457.0924.

^{1}H NMR (300 MHz, $CDCl_3$). δ (ppm) 8.81 (1H, d, J = 8.29 Hz); 8.49 (1H, s); 8.02 (1H, d, J = 7.25 Hz); 2.52 (6H, s); 1.69 (15H, s).

^{13}C NMR (75 MHz, $CDCl_3$). δ (ppm) 152.45, 150.70, 141.49, 138.72, 124.67, 97.08, 96.68, 18.88, 9.29.

Cp*Rh (*N*-(1,10-phenanthrolin-5-yl)acetamide) chloride, [Cp*Rh(H-L2)Cl]Cl.

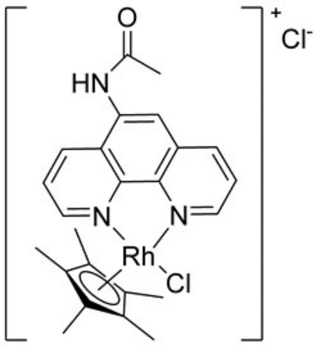

Working under nitrogen, 105 mg (0.17 mmol, 1 eq) of pentamethylcyclopentadienyl rhodium(III) chloride dimer was suspended in 2 mL methanol. To this suspension, a solution of 81 mg (0.34 mmol, 2 eq) of N-(1,10-phenanthrolin-5-yl)acetamide in 1 mL methanol was added. The mixture immediately turned into a yellow/orange clear solution, indicating binding of Rh to the phenenthrolin nitrogen atoms. The product was precipitated by addition of diethylether. Total yield: 128 mg (69%).

Mass spectrometry: $C_{24}H_{26}ClN_3ORh$ (M+) obs 510.0837, calc 510.0814.

^{1}H NMR (400 MHz, $CDCl_3$). δ 9.78 (1H, d J= 8.48 Hz, Haryl); 9.14 (1H, d J=4.97 Hz, Haryl); 9.07 (1H, d J=4.68, Haryl); 8.48 (1H, s, H6); 8.37 (1H, d J=8.04 Hz, Haryl); 8.03 (1H, dd J=8.48 5.12 Hz, Haryl); 7.91 (1H, dd J= 8.18 5.12, Haryl), 2.57 (3H, s, acetamide CH_3); 1.70 (15H, s, Cp* CH_3) ppm.

^{13}C NMR (400 MHz, $CDCl_3$). δ 171.96 (C=O); 151.42-149.85-145.58-142.81-138.30-137.51-135.19-130.80-127.42-126.76-126.38-118.89 (Caryl); 97.17-97.08 (Cp* ring); 24.67 (acetamide CH_3); 9.35 (Cp* CH_3) ppm.

3. Expression, purification and characterization of TbADH variants

The gene encoding for wild-type TbADH with *N*-terminal StrepTagII, cloned in the expression vector pET21a, was obtained from Biomatik (Ontario, Canada). Mutagenesis was performed using the QuikChange Lightning Site-Directed Mutagenesis Kit (Agilent Technologies), following manufacturer's instructions. The wild-type TbADH sequence and primers used for mutagenesis are detailed below. Recombinant overexpression was carried out in *E. coli* BL21(DE3). Briefly, bacteria were grown in LB medium (10 g/L tryptone, 5 g/L yeast extract, 10 g/L NaCl) supplemented with 100 μg/mL carbenicillin. Cultures were grown in baffled flasks at 37 °C and 200 rpm until the OD_{600} reached ~0.8. Subsequently, $ZnCl_2$ was added (final concentration 1 mM) and the expression of TbADH gene was induced with isopropyl β-D-1-thiogalactopyranoside (IPTG; final concentration 1 mM). Cultures were incubated at 30 °C and 120 rpm for further 20-22 h and then harvested. Cells were resuspended in 100 mM TrisHCl, 150 mM NaCl, pH 8 supplemented with 1 mg/mL lysozyme, cOmplete™ protease inhibitor (Roche) and Benzonase® endonuclease (Merck). After stirring 1 hour on ice, cells were lysed by sonication (Fisher Scientific FB-120) and centrifuged (10 °C, 15 min, 75000 *g*). The soluble fraction was heat treated at 60 °C for 15 min, centrifuged again and then purified by affinity chromatography using a 5 mL StrepTrap HP column (GE Healthcare), following manufacturer's instructions. From 0.5 L lab-scale fermentation, approximately 35-50 mg TbADH were obtained (Table S1). Purity was routinely assessed by SDS-PAGE followed by Coomassie staining. Protein concentration was determined by the Bradford assay, using bovine serum albumin as standard.

Table S1. Characteristics of the purified recombinant TbADH variants.

	Specific activity (U / mg)	Free thiols : monomer	Zn atoms : monomer	Protein yield (mg protein / 0.5L culture)
WT TbADH	73.71 ± 7.09	0.98 ± 0.003	0.64 ± 0.02	45-50
TbADH 3M	87.73 ± 6.91	0.00 ± 0.010	0.68 ± 0.01	35-41
TbADH 5M	0.04 ± 0.01	0.72 ± 0.030	0.01 ± 0.01	40-52

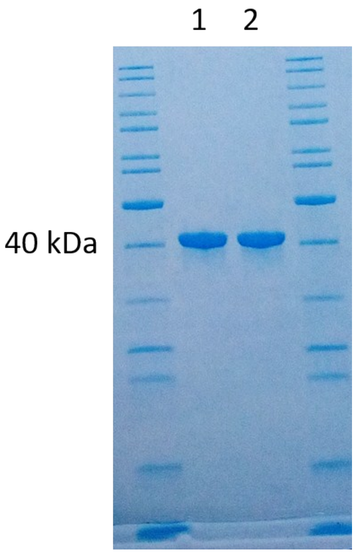

Figure S1. SDS-PAGE analysis of the purified TbADH mutants 3M and 5M used in this study. Lane 1: TbADH 3M (C203S-C283A-C295A); lane 2: TbADH 5M (H59A-D150A-C203S-C283A-C295A). Proteins were loaded at 2 μg / lane.

Enzyme activity. The native activity was assayed as previously described, by measuring $NADP^+$ reduction coupled to racemic 2-butanol oxidation.[4] The reaction mixture contained 100 mM TrisHCl pH 7.8, 0.5 mM $NADP^+$ and 150 mM *rac*-2-butanol. After warming to 40 °C, and the reaction was started by the addition of 25 nM enzyme. Increase in absorbance at 340 nm was monitored with a Shimadzu UV-2600 spectrophotometer, equipped with CPS-100 temperature controller (Figure S2).

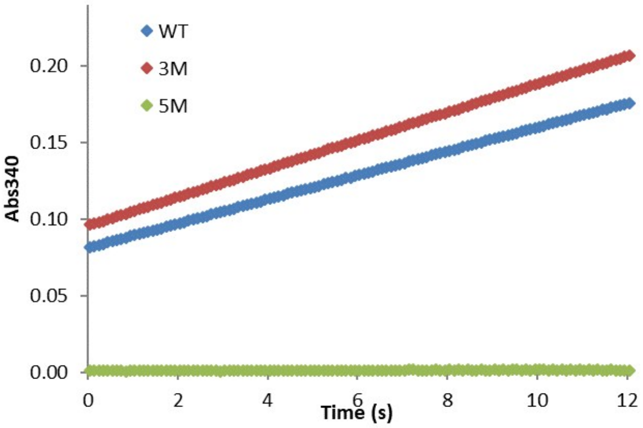

Figure S2. Enzymatic activity assays for WT TbADH, TbADH 3M and TbADH 5M.

Ellman assay. The free thiol content was determined using Ellman's reagent, following the manufacturer's instructions (Thermo Scientific). Reaction buffer (0.1 M sodium phosphate, 1 mM EDTA, pH 8.0, 725 µL) was mixed with 15 µL of reagent solution (4 mg/mL 5,5'-dithio-bis(2-nitrobenzoic acid)) and 35 µL sample in a 1 cm cuvette. After 20 min incubation, the absorbance was measured at 412 nm, and the free thiol concentration was determined from the difference signal between the sample and a control containing no protein, using ε = 14150 M^{-1} cm^{-1} and correcting by the dilution factor.

ICP-MS. Metal (Zn and Rh) content was determined by inductively coupled plasma mass spectrometry (ICP-MS) with a Thermo Fisher iCAP-Q instrument, after digestion of the protein in nitric acid (trace metal grade, Fisher Scientific) as previously described.[5]

ESI-TOF. Mass spectrometry analysis was performed by electrospray ionisation time-of-flight (ESI-TOF) in a Bruker Impact II spectrometer. Before analysis, the protein samples were desalted by PD-10 in pure water, concentrated up to ~5 mg mL^{-1} and then mixed with 1 volume of 0.1% formic acid in acetonitrile.

Characterisation of TbADH 5M by ESI-TOF mass spectrometry

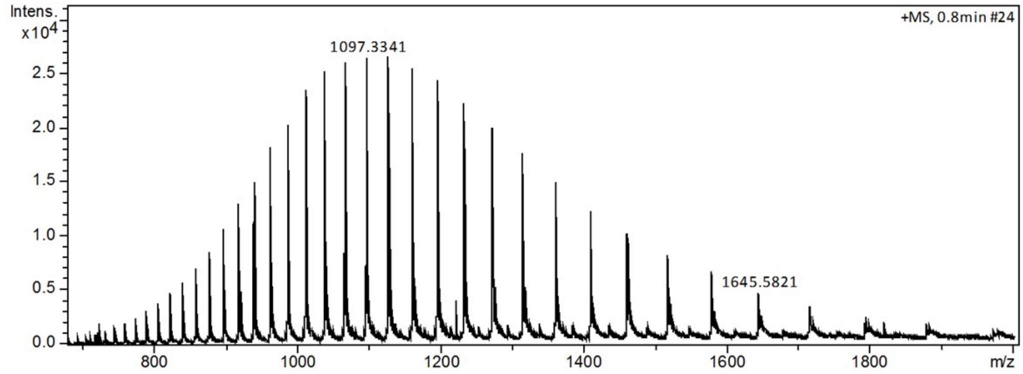

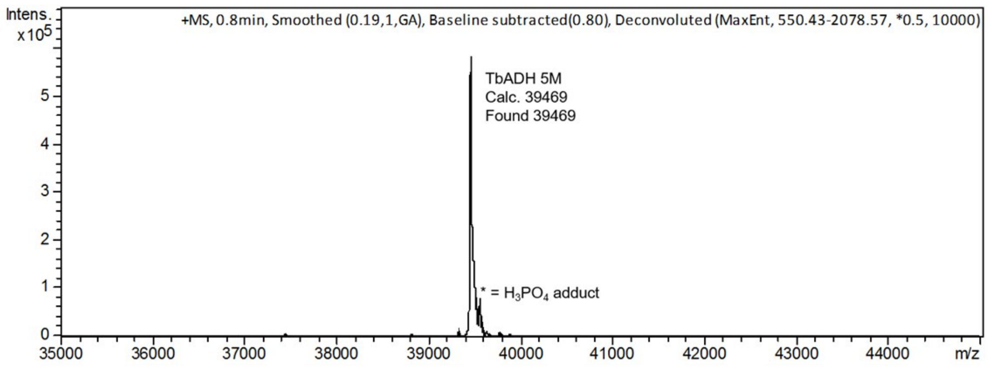

<u>TbADH WT sequences</u>

>Gene sequence

ATGTGGAGCCACCCGCAGTTCGAAAAATCTTCTGGTCTGGTTCCGCGTGGATCCATGAAGGGCTTCGC

GATGCTGAGCATTGGTAAGGTTGGCTGGATCGAGAAAGAAAAACCGGCTCCGGGTCCGTTCGACGCTA

TCGTGCGTCCGCTGGCTGTTGCGCCGTGCACCTCTGACATCCATACCGTTTTCGAGGGTGCGATCGGTG

AGCGCCACAACATGATCCTGGGTCACGAAGCGGTGGGCGAGGTTGTTGAAGTTGGCTCTGAGGTGAAA

GACTTTAAGCCGGGTGATCGTGTTGTTGTTCCGGCAATCACCCCGGACTGGCGTACTTCCGAAGTTCA

GCGCGGCTACCACCAGCACTCTGGCGGTATGCTGGCGGGTTGGAAATTCTCCAACGTTAAGGACGGCG

TGTTCGGCGAGTTCTTCCATGTGAACGACGCTGACATGAACCTGGCGCACCTGCCGAAGGAAATCCCG

CTGGAAGCGGCGGTTATGATTCCGGATATGATGACTACTGGTTTCCATGGTGCAGAGCTGGCAGACAT

TGAGCTGGGTGCAACCGTTGCGGTTCTGGGTATCGGTCCGGTTGGCCTGATGGCGGTGGCTGGTGCGA

AACTGCGTGGTGCGGGTCGTATCATCGCGGTTGGTTCTCGTCCGGTGTGCGTTGATGCTGCTAAATAC

TACGGTGCGACCGACATTGTTAACTACAAAGACGGTCCGATCGAATCTCAGATTATGAACCTGACCGA

GGGCAAAGGCGTGGACGCAGCGATTATCGCAGGTGGTAACGCGGATATCATGGCGACCGCTGTTAAAA

TCGTTAAACCGGGTGGTACTATTGCGAACGTGAACTATTTCGGTGAAGGCGAAGTGCTGCCGGTGCCG

CGTCTGGAATGGGGTTGTGGTATGGCACATAAGACCATTAAAGGTGGTCTGTGTCCGGGCGGTCGTCT

GCGCATGGAACGTCTGATTGACCTGGTTTTCTACAAACGTGTTGACCCGTCTAAACTGGTTACCCACG

TGTTCCGTGGTTTCGACAACATCGAAAAGGCTTTTATGCTGATGAAAGATAAACCGAAAGATCTGATC

AAACCGGTGGTTATCCTGGCGTAA

>Protein sequence (the mutation sites are highlighted in red: H59, D150, C203, C283, C295 – numbering according to the WT TbADH sequence; the *N*-term StrepTagII and the thrombin cleavage site peptide sequence are highlighted in blue).

MWSHPQFEKSSGLVPRGSMKGFAMLSIGKVGWIEKEKPAPGPFDAIVRPLAVAPCTSDIHTVFEGAIGE

RHNMILG**H**EAVGEVVEVGSEVKDFKPGDRVVVPAITPDWRTSEVQRGYHQHSGGMLAGWKFSNVKD

GVFGEFFHVNDADMNLAHLPKEIPLEAAVMIP**D**MMTTGFHGAELADIELGATVAVLGIGPVGLMAVAG

AKLRGAGRIIAVGSRPV**C**VDAAKYYGATDIVNYKDGPIESQIMNLTEGKGVDAAIIAGGNADIMATAVKI

VKPGGTIANVNYFGEGEVLPVPRLEWGCGMAHKTIKGGLCPGGRLRMERLIDLVFYKRVDPSKLVTHV

FRGFDNIEKAFMLMKDKPKDLIKPVVILA

TbADH mutagenesis primers

Mutant	Primer sequences
H59A	fwd: 5'-GCCACAACATGATCCTGGGTGCCGAAGCGGTGG<br>rev: 5'-CCACCGCTTCGGCACCCAGGATCATGTTGTGGC
D150A	fwd: 5'-GGCGGTTATGATTCCGGCTATGATGACTACTGGTT<br>rev: 5'-AACCAGTAGTCATCATAGCCGGAATCATAACCGCC
C203S	fwd: 5'-GTTCTCGTCCGGTGAGCGTTGATGCTGCT<br>rev: 5'-AGCAGCATCAACGCTCACCGGACGAGAAC
C283A	fwd: 5'-CCGCGTCTGGAATGGGGTGCTGGTATGGCACAT<br>rev: 5'-ATGTGCCATACCAGCACCCCATTCCAGACGCGG
C295A	fwd: 5'-CATTAAAGGTGGTCTGGCTCCGGGCGGTCGTCTG<br>rev: 5'-CAGACGACCGCCCGGAGCCAGACCACCTTTAATG

4. Bioconjugation of ligands and complexes to TbADH 5M

4.1 Covalent conjugation of brominated ligands to TbADH 5M (5M-C37L1 and 5M-C37L2).

TbADH 5M (4-6 mg mL^{-1}, 100-150 μM) and brominated ligand Br-**L1** or Br-**L2** (4 equiv. from a 25 mM stock in acetonitrile/buffer or acetonitrile) were mixed in buffer (Tris-HCl or sodium phosphate, 100 mM, 0.2 – 1 mL final volume) and allowed to react at room temperature for 1 h. After this time, the excess of the labelling agent was removed by desalting on a PD10 column, and the labelled protein was concentrated by Vivaspin (10 kDa MWCO) up to 4-6 mg mL^{-1}.

*Characterisation of 5M-C37**L1** and 5M-C37**L2** by Ellman assay*

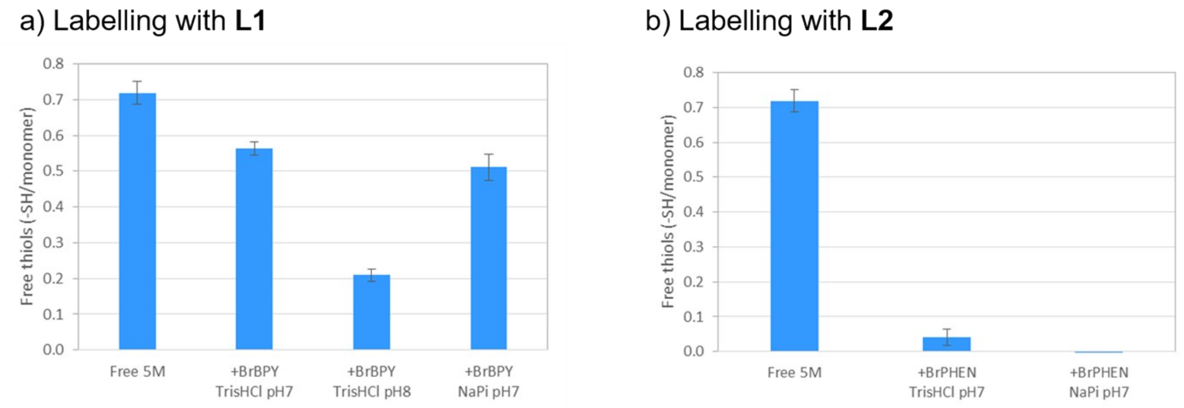

Figure S3. Free thiol content in 5M-C37**L1** and 5M-C37**L2,** determined by Ellman assay. a) covalent modification with ligand Br-**L1** in different buffers and pHs; b) covalent modification with ligand Br-**L2** in different buffers at pH 7.

*Characterisation of 5M-C37**L1** and 5M-C37**L2** by ESI-TOF mass spectrometry*

<u>5M-C37**L1** TrisHCl pH 7</u>

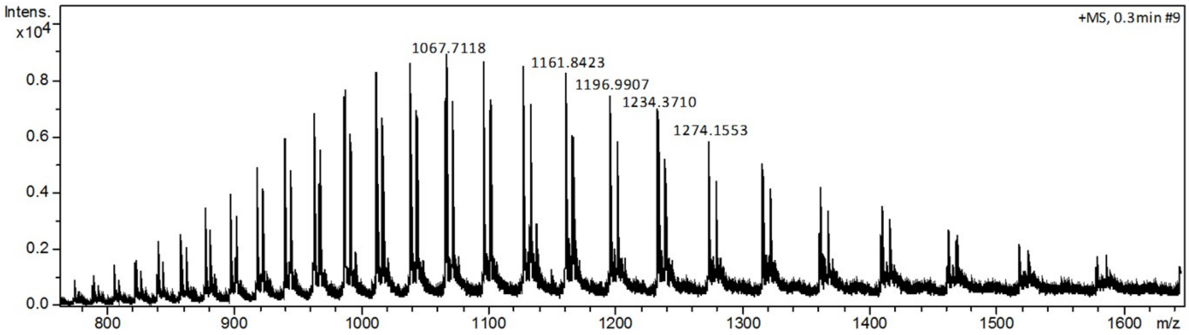

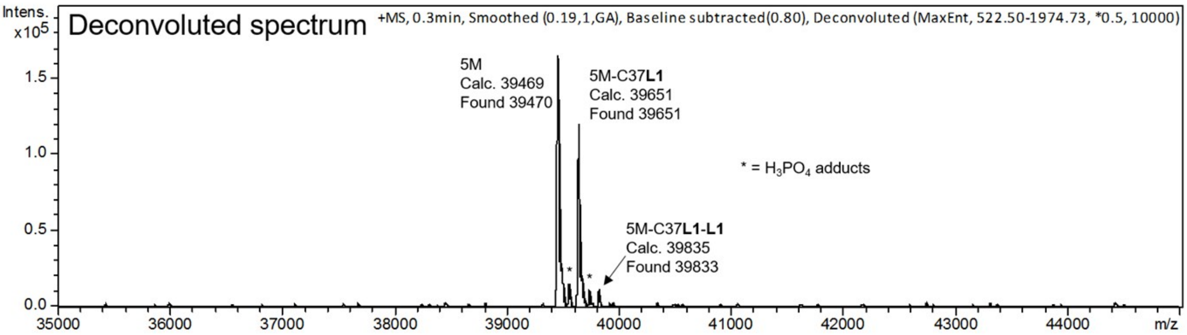

<u>5M-C37**L1** TrisHCl pH 8</u>

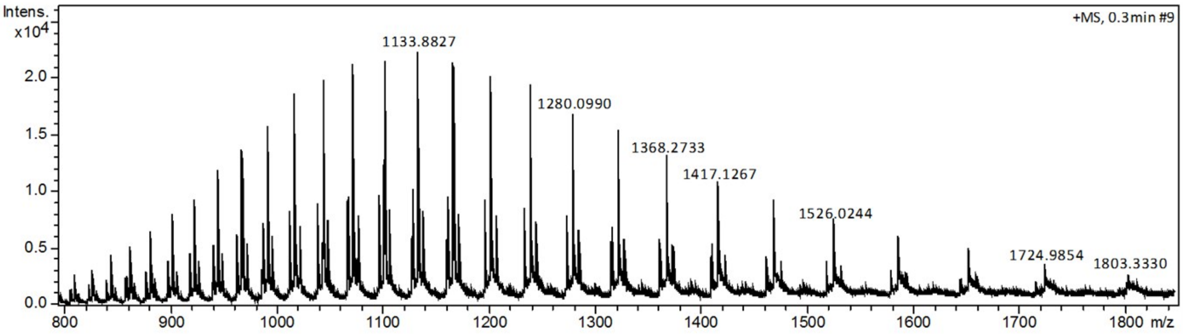

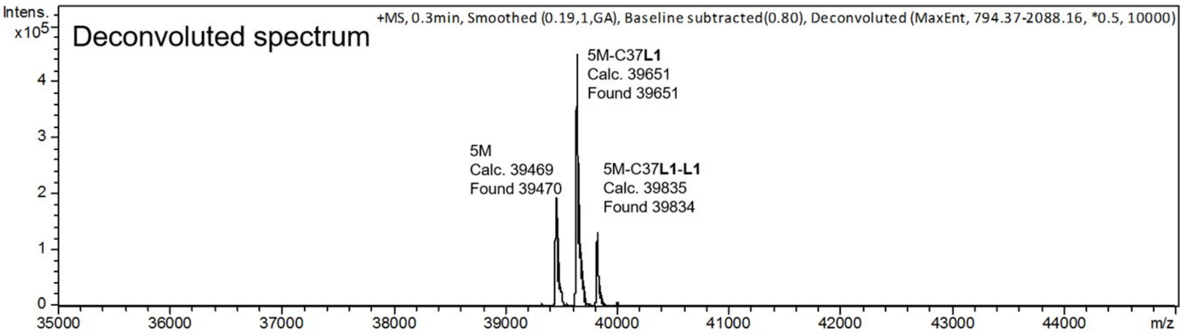

5M-C37**L1** Sodium phosphate pH 7

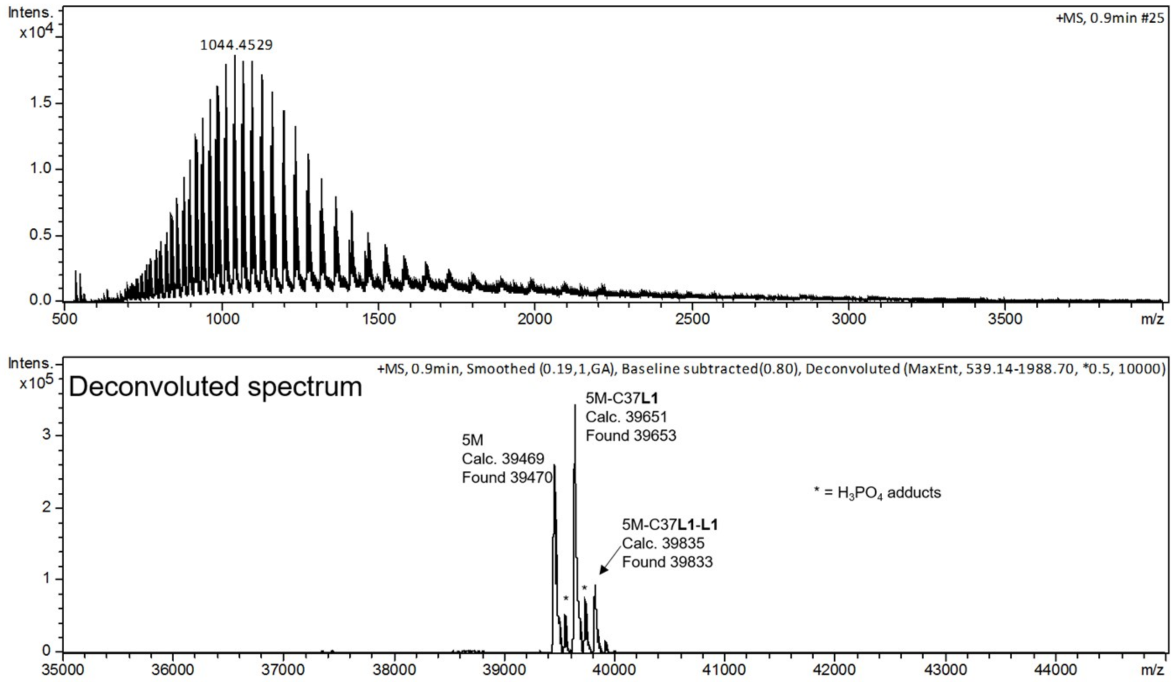

5M-C37**L2** TrisHCl pH 7

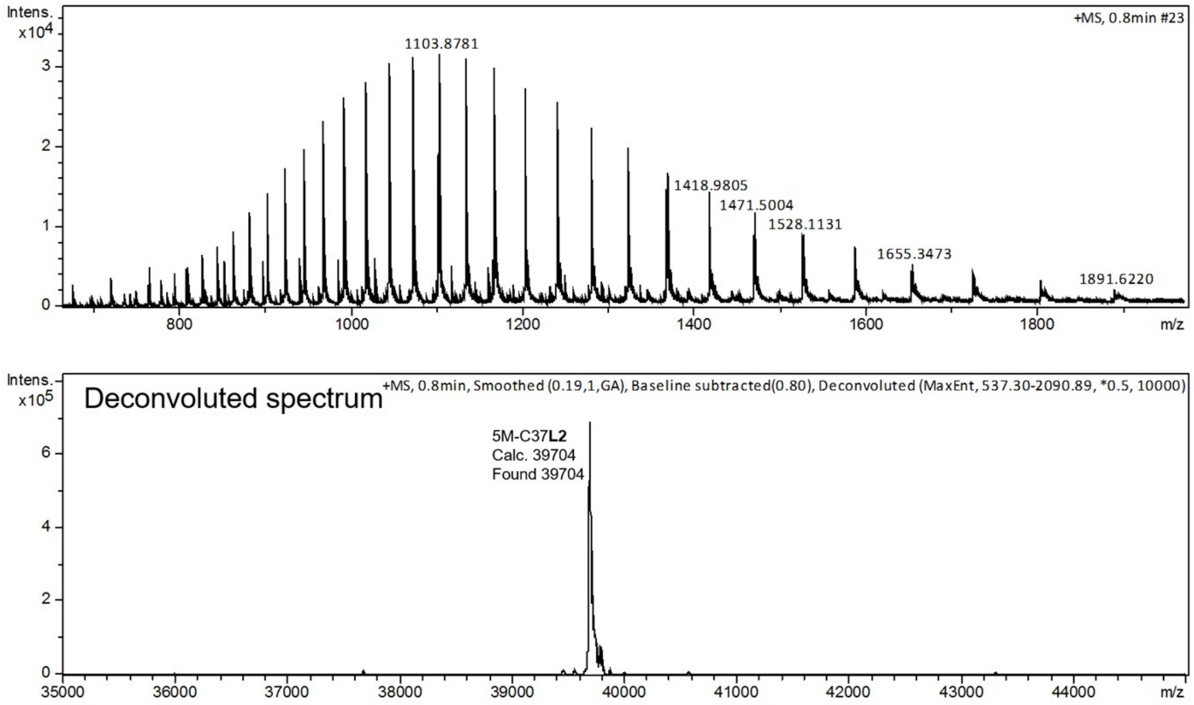

5M-C37**L2** Sodium phosphate pH 7

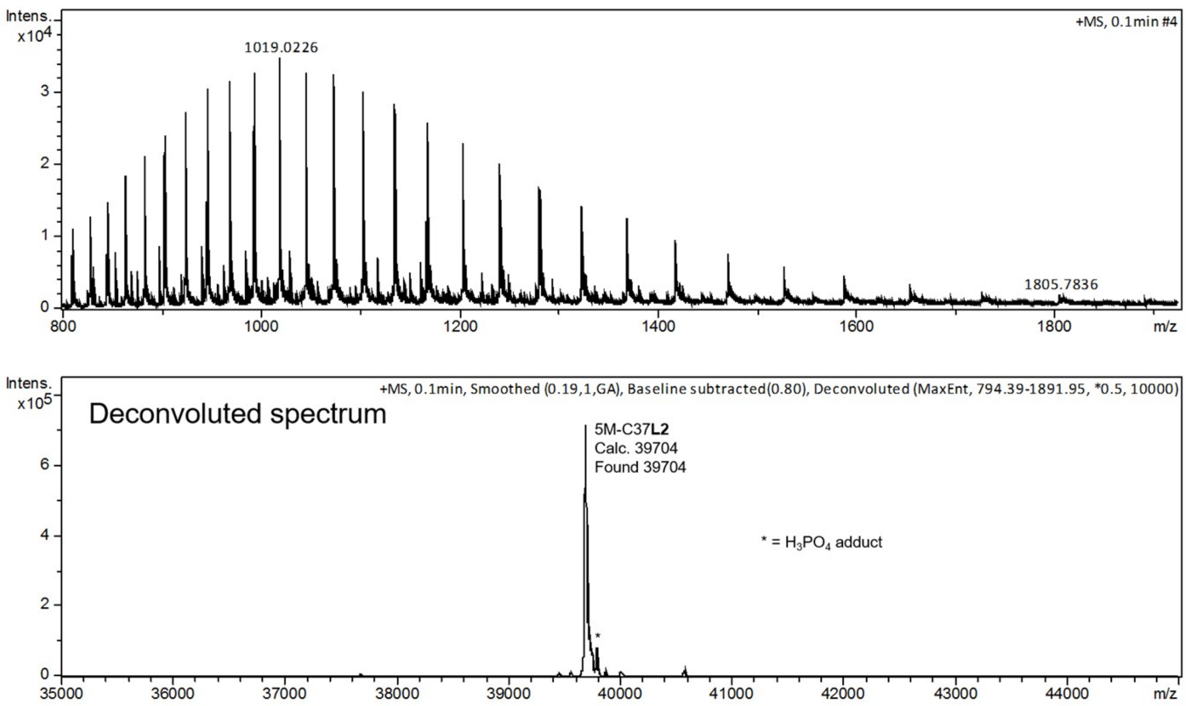

*Characterisation of 5M-C37**L1** and 5M-C37**L2** by UV-Vis spectrscopy*

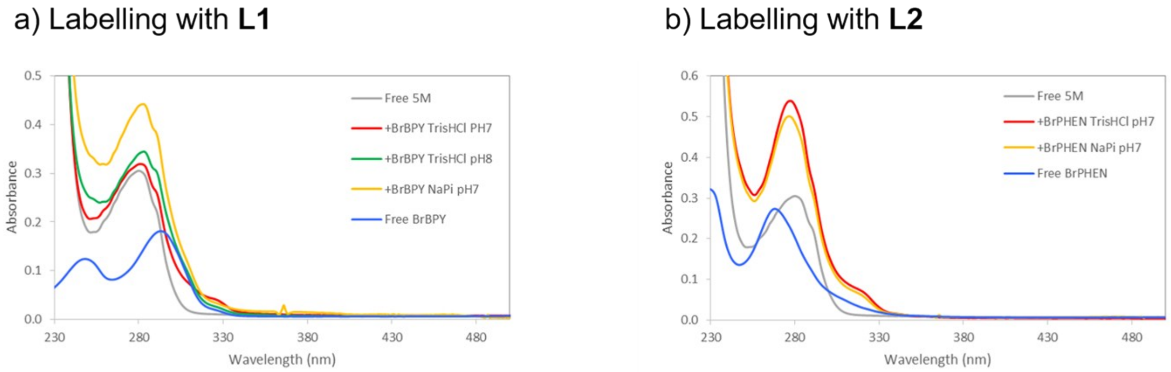

Figure S4. UV-Vis spectra of covalently modified TbADH 5M with brominated ligands Br-**L1** (a) and Br-**L2** (b).

4.2 Covalent conjugation of brominated rhodium complexes to TbADH 5M ([Cp*Rh(5M-C37L1)] and [Cp*Rh(5M-C37L2)]).

TbADH 5M (4-6 mg mL^{-1}, 100-150 μM in buffer, 100 mM) and the brominated complex [Cp*Rh(Br-**L1**)Cl]Cl or [Cp*Rh(Br-**L2**)Cl]Cl (4 equiv. from a 25 mM stock in acetonitrile / buffer) were mixed (0.2 – 1 mL final volume) and allowed to react at room temperature for 1 h. After this time, the excess of the labelling agent was removed by desalting on a PD10 column, and the labelled protein was concentrated by Vivaspin (10 kDa MWCO) up to 10-12 mg mL^{-1}. The following analyses were performed: Ellman assay, protein concentration by Bradford assay, UV-Vis spectroscopy, ICP-MS, ESI-TOF, and MALDI-TOF of the peptide mixture, following chymotrypsin digestion.

*Characterisation of [Cp*Rh(5M-C37**L1**)] and [Cp*Rh(5M-C37**L2**)] by Ellman assay*

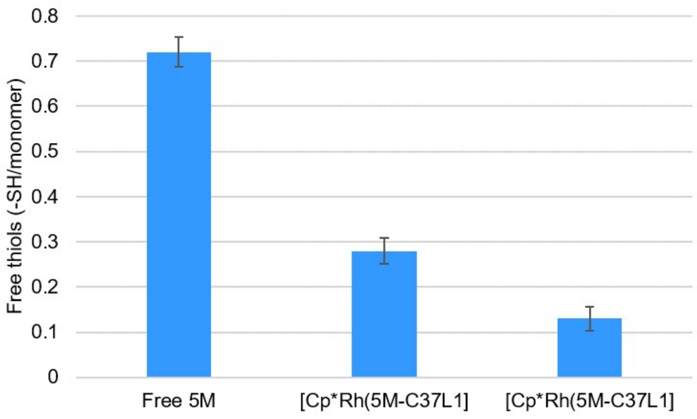

Figure S5. Free thiol content after covalent modification of TbADH 5M with complexes [Cp*Rh(Br-**L1**)Cl]Cl and [Cp*Rh(Br-**L2**)Cl]Cl in 100 mM TrisHCl, pH 7.

*Characterisation of [Cp*Rh(5M-C37**L1**)] and [Cp*Rh(5M-C37**L2**)] by ESI-TOF mass spectrometry*

[Cp*Rh(5M-C37**L1**)] TrisHCl pH 7

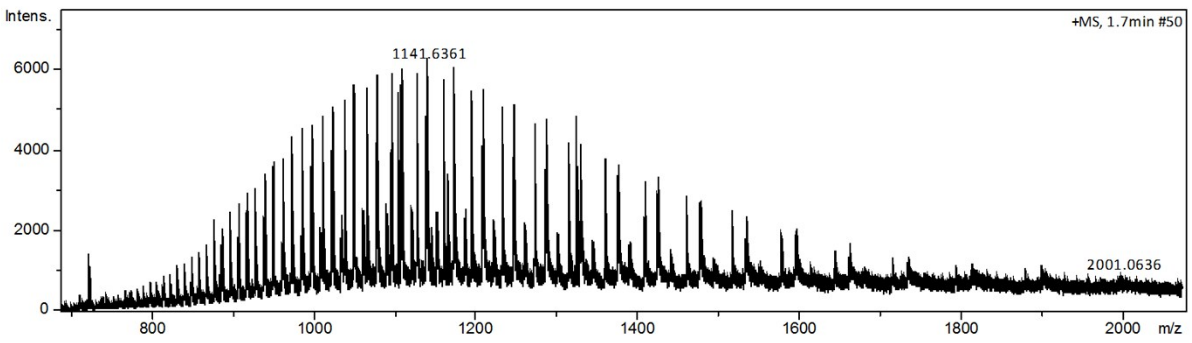

[Cp*Rh(5M-C37**L1**)] TrisHCl pH 8

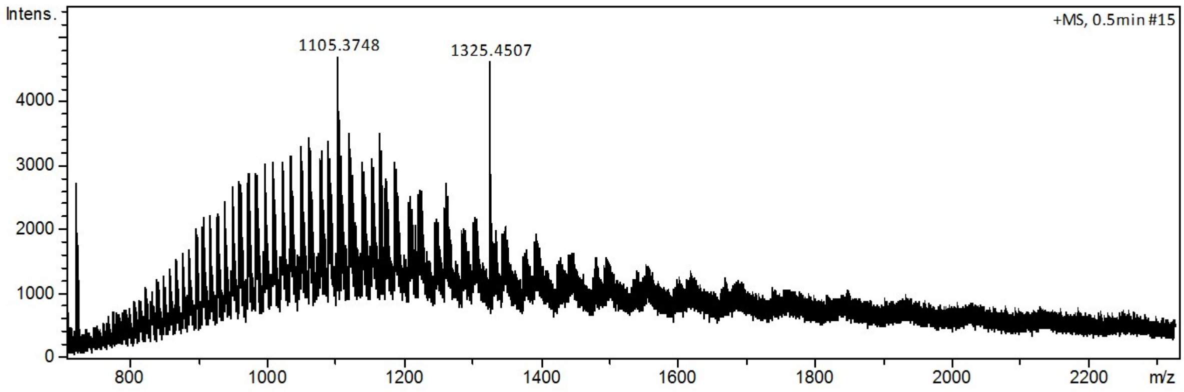

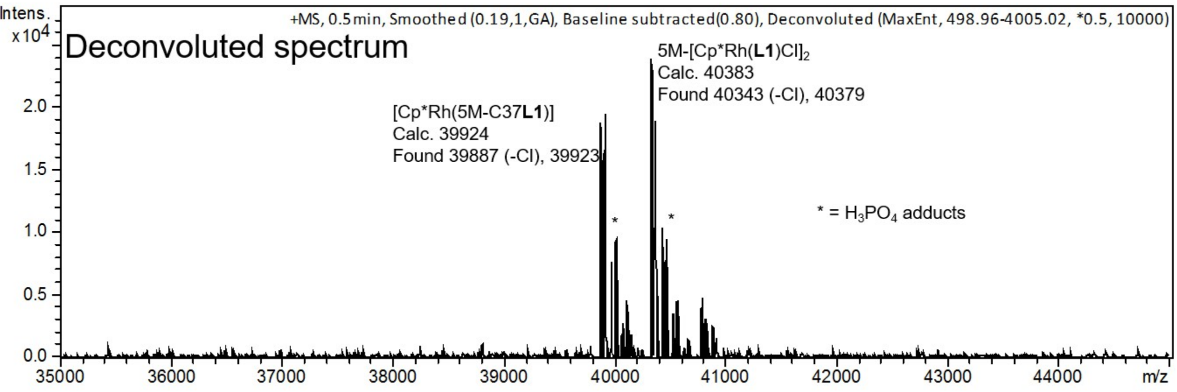

[Cp*Rh(5M-C37**L1**)] TrisHCl pH 9

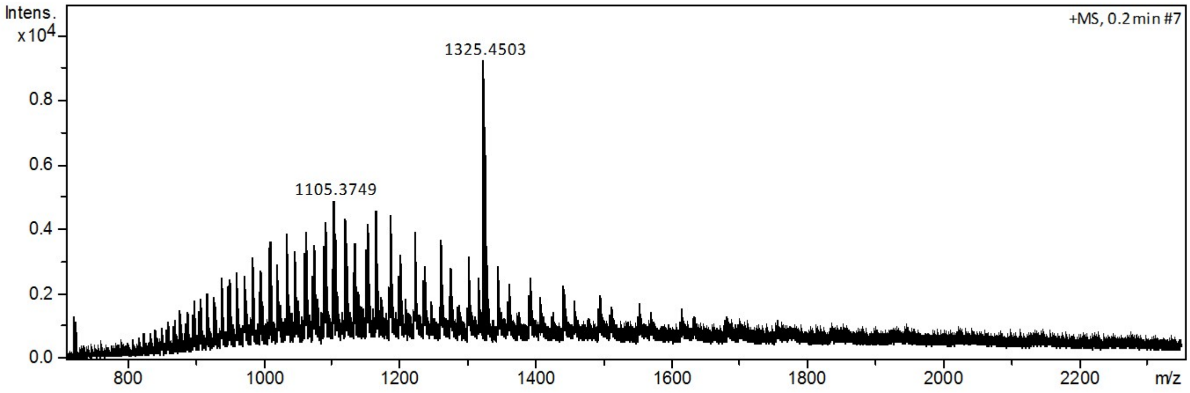

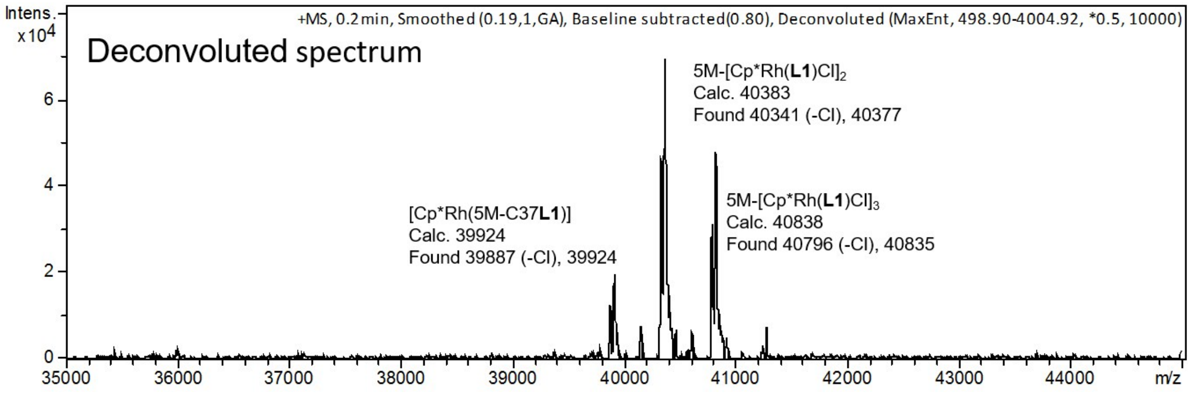

[Cp*Rh(5M-C37**L2**)] TrisHCl pH 7

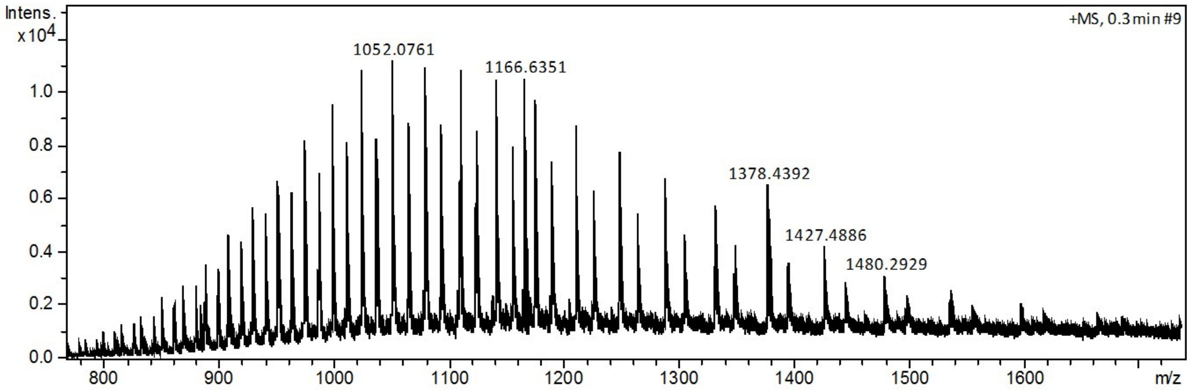

*Digestion of [Cp*Rh(5M-C37**L1**)] and [Cp*Rh(5M-C37**L2**)] by chymotrypsin*[6]

To confirm the labelling site, the modified protein samples were treated with 6M urea and 10 mM dithiothreitol (DTT) for 1 h at 60 °C ,and subsequently alkylated with 40 mM iodoacetamide (IAA). Excess IAA was neutralised with extra DTT and samples were digested with chymotrypsin (from bovine pancreas, sequencing grade, Roche) in 100 mM TrisHCl, 10 mM $CaCl_2$ pH 7.8 for 24

h at 25 °C. The peptides were purified by cleanup C18 tips (Agilent Technologies), and analysed by matrix-assisted laser desorption/ionisation (MALDI-TOF) with a Bruker UltraFlex III spectrometer. α-Cyano-4-hydroxycinnamic acid (CHCA) was used as matrix.

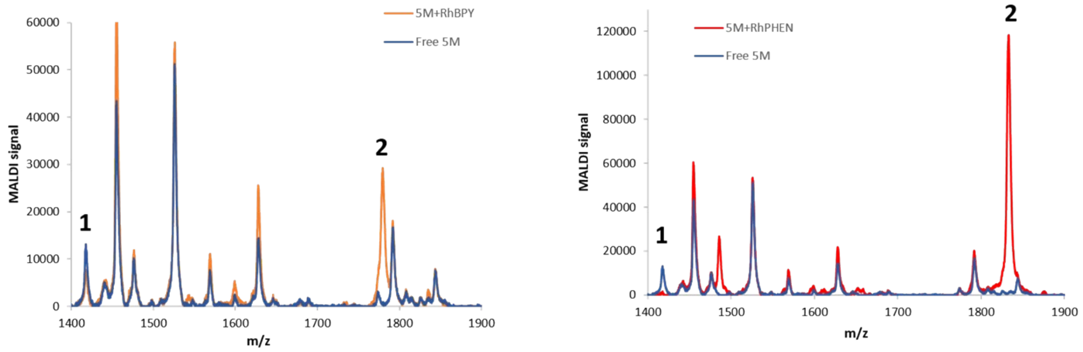

Peak label	Peptide: AVAPCTSDIHTVF; $M_{peptide}$ = 1360.7	
1	Free 5M	1417.6776 ($M_{peptide}$ + iodoacetamide)
2	[Cp*Rh(5M-C37**L1**)]	1779.6487 ($M_{peptide}$ + [Cp*Rh**L1**])

Peak label	Peptide: AVAPCTSDIHTVF; $M_{peptide}$ = 1360.7	
1	Free 5M	1417.6780 ($M_{peptide}$ + iodoacetamide)
2	[Cp*Rh(5M-C37**L2**)]	1833.0064 ($M_{peptide}$ + [Cp*Rh**L2**])

Figure S6. MALDI-TOF analysis of the peptide mixture obtained by chymotrypsin digestion of TbADH 5M covalently modified with rhodium complexes (left: modification with [Cp*Rh(Br-**L1**)]; right: modification with [Cp*Rh(Br-**L2**)]).

5. Formate dehydrogenase activity of artificial metalloenzymes

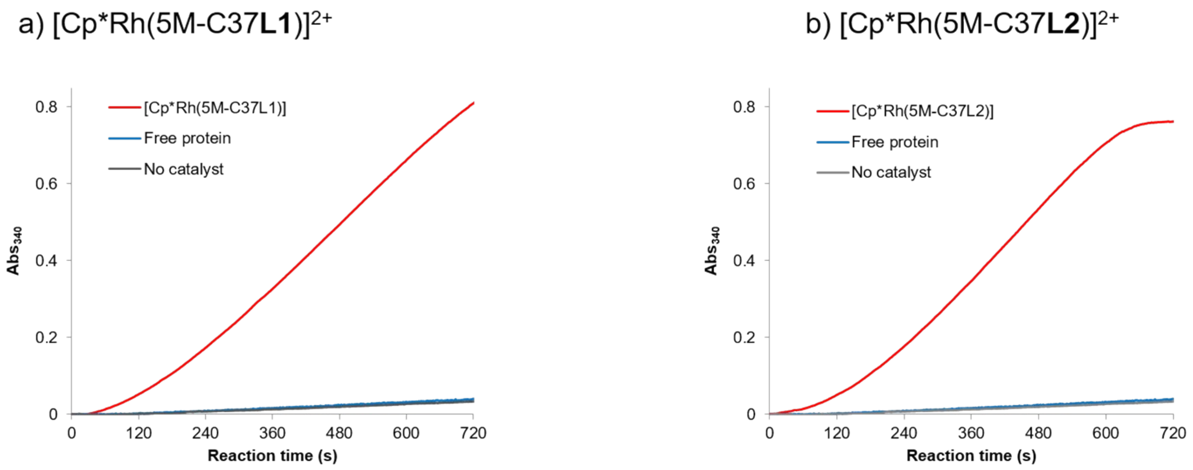

Figure S7. Time course for the reduction of NADP$^+$ by the artificial metalloenzymes**:** a) [Cp*Rh(5M-C37**L1**]$^{2+}$ and b) [Cp*Rh(5M-C37**L2**]$^{2+}$. Reaction conditions: 100 mM sodium phosphate buffer pH 7, sodium formate (500 mM), NADP$^+$ (0.15 mM), artificial metalloenzyme (12.5 μM Rh), 50 °C.

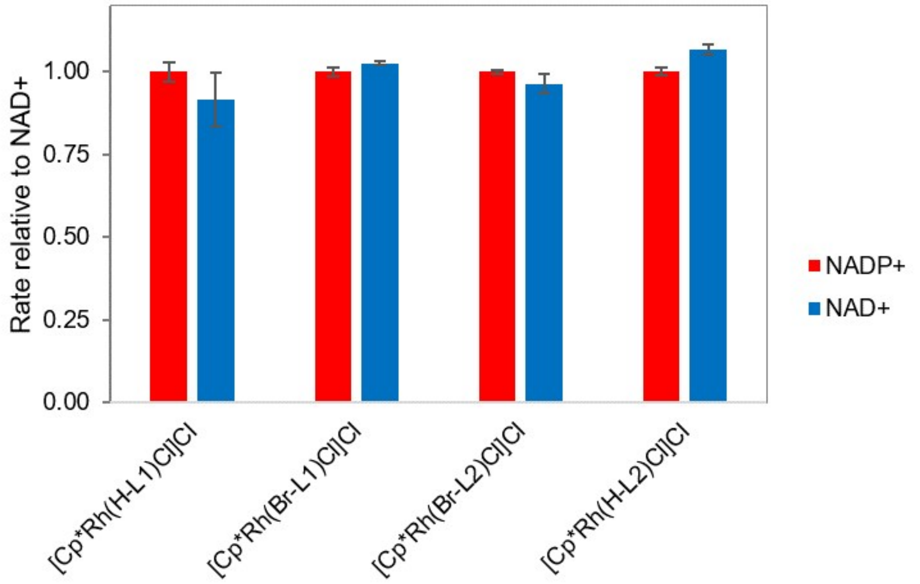

Figure S8. Relative rates of NAD$^+$ and NADP$^+$ reduction by free brominated and non-brominated rhodium catalysts. Catalysis conditions: 100 mM sodium phosphate buffer pH 7, sodium formate (500 mM), NADP$^+$ (0.42 mM), rhodium catalyst (25 μM), 50 °C.

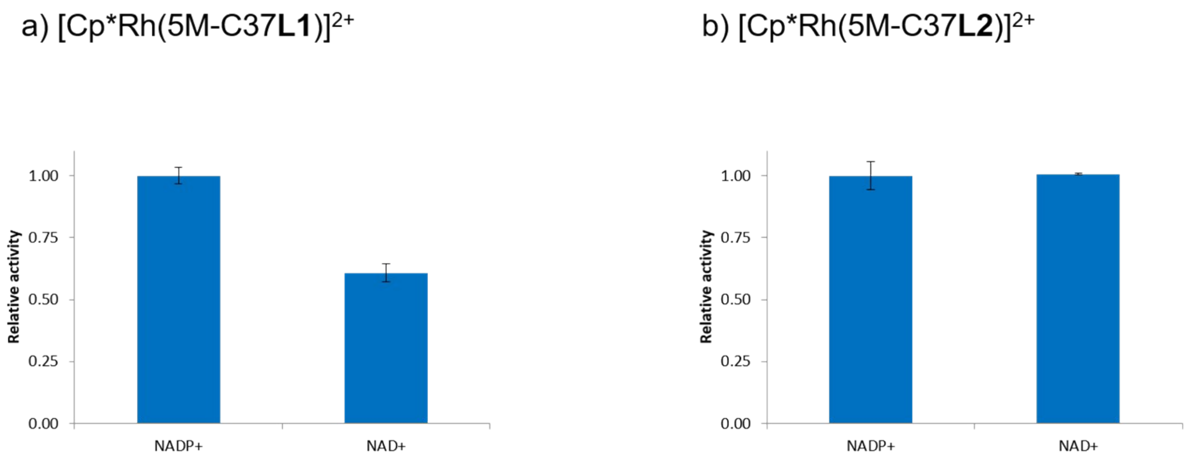

Figure S9. Relative rates of NAD$^+$ and NADP$^+$ reduction by artificial metalloenzymes [Cp*Rh(5M-C37**L1**)]$^{2+}$ and [Cp*Rh(5M-C37**L2**)]$^{2+}$. Catalysis conditions: 100 mM sodium phosphate buffer pH 7, sodium formate (500 mM), NADP$^+$ (0.15 mM), rhodium catalyst (12.5 μM determined by ICP-MS), 50 °C.

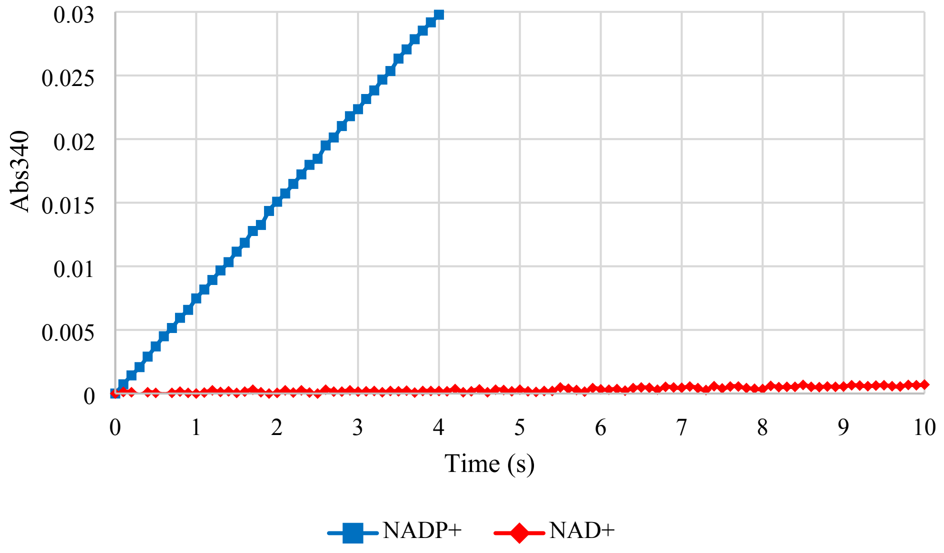

Figure S10. Cofactor specificity of WT TbADH. Assays were performed in a 1 cm cuvette and followed by UV-Vis spectroscopy, by monitoring the absorbance at 340 nm. Conditions: 100 mM TrisHCl buffer pH 7.8, NAD(P)$^+$ (0.5 mM), 2-butanol (150 mM), WT TbADH (25 nM), 40 °C. Calculated specific activities were: 73 U mg^{-1} with NADP$^+$ and 0.6 U mg^{-1} with NAD$^+$.

6. Compatibility between Rh catalysts and TbADH

(A)

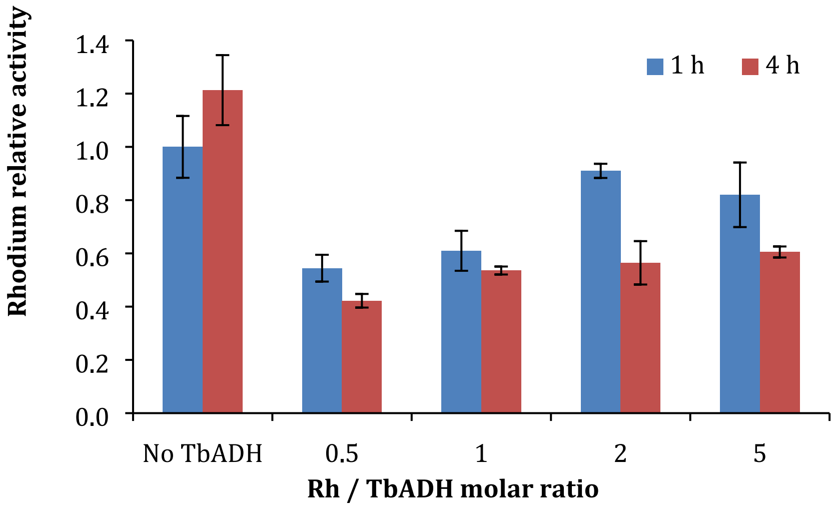

(B)

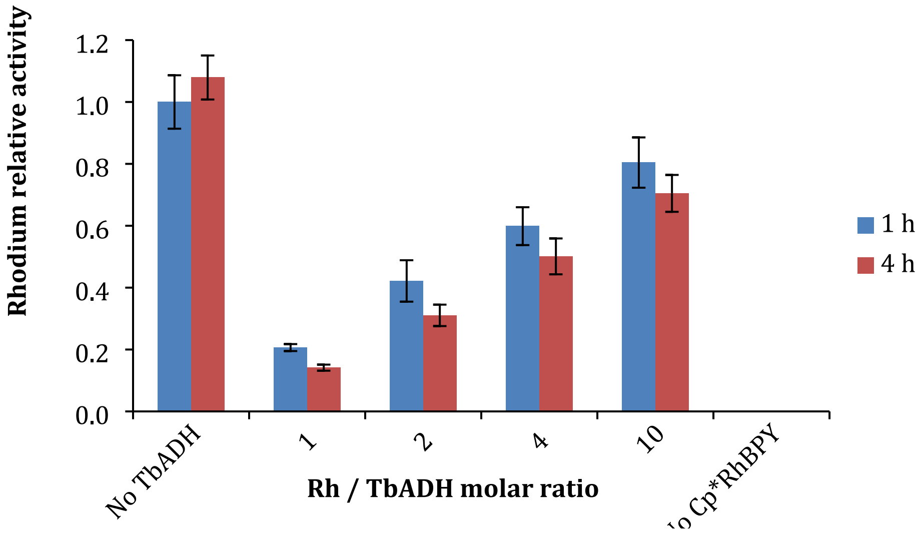

Figure S11. Relative activity of Rh catalysts after incubation with WT TbADH at different molar ratios. (A) [Cp*Rh(5M-C37**L1**)]$^{2+}$; (B) [Cp*Rh(H-**L1**)Cl]Cl. Rhodium catalysts (10 μM for bioconjugated catalysts, 20 μM for free catalysts) were incubated with WT TbADH (2 μM, 5 μM, 10 μM, 20 μM) in sodium phosphate buffer (100 mM, pH 7) at 25 °C for 1 h and 4 h, respectively. Catalysis conditions: 100 mM sodium phosphate buffer pH 7, sodium formate (100 mM), NADP^{+} (0.15 mM), 50 °C.

(A)

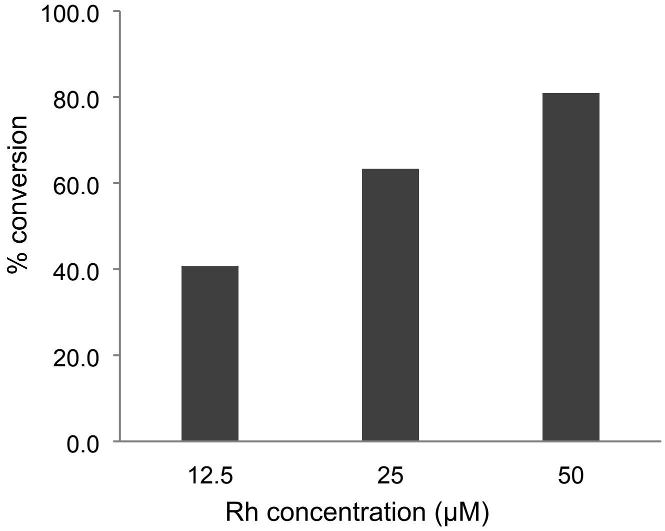

(B)

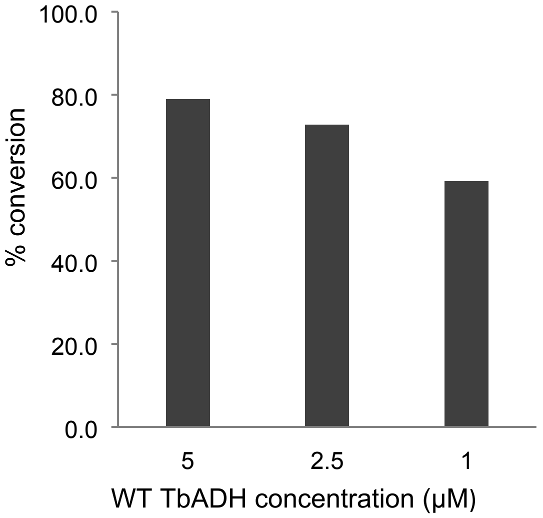

(C)

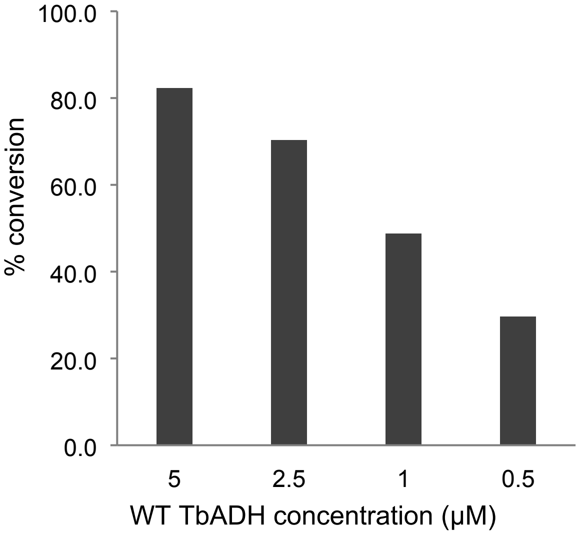

Figure S12. Reduction of 4-phenyl-2-butanone using TbADH and Rh catalysts in different concentrations**.** (A) Effect of $[Cp^*Rh(5M\text{-}C37\mathbf{L1})]^{2+}$ concentration using 5 µM WT TbADH; (B) Effect of WT TbADH concentration using 25 µM $[Cp^*Rh(5M\text{-}C37\mathbf{L1})]^{2+}$; (C) Effect of WT TbADH concentration using 25 µM [Cp*Rh(H-**L1**)Cl]Cl. Conversions were determined by GC-FID.

7. References

1. B. I. Ipe, K. Yoosaf and K. G. Thomas, *J. Am. Chem. Soc.*, 2006, **128**, 1907-1913.
2. J. Bos, F. Fusetti, A. J. M. Driessen and G. Roelfes, *Angew. Chem., Int. Ed.*, 2012, **51**, 7472-7475.
3. F. Hollmann, B. Witholt and A. Schmid, *J. Mol. Catal. B: Enzym.*, 2002, **19-20**, 167-176.
4. R. J. Lamed and J. G. Zeikus, *Biochem. J.*, 1981, **195**, 183-190.
5. C. A. McDevitt, A. D. Ogunniyi, E. Valkov, M. C. Lawrence, B. Kobe, A. G. McEwan and J. C. Paton, *PLoS Pathog.*, 2011, **7**, e1002357.
6. T. W. Jaskolla, D. G. Papasotiriou and M. Karas, *Journal of Proteome Research*, 2009, **8**, 3588-3597.
